# B7-H3 as a therapeutic target in advanced prostate cancer

**DOI:** 10.1016/j.eururo.2022.09.004

**Published:** 2022-09-13

**Authors:** Christina Guo, Ines Figueiredo, Bora Gurel, Antje Neeb, George Seed, Mateus Crespo, Suzanne Carreira, Jan Rekowski, Jon Welti, Lorenzo Buroni, Denisa Bogdan, Lewis Gallagher, Adam Sharp, Maria D. Fenor de la Maza, Pasquale Rescigno, Daniel Westaby, Khobe Chandran, Ruth Riisnaes, Ana Ferreira, Susana Miranda, Bianca Calì, Andrea Alimonti, Silvia Bressan, Alana H. T. Nguyen, Michael M. Shen, Jessica E. Hawley, Aleksandar Obradovic, Charles G. Drake, Claudia Bertan, Chloe Baker, Nina Tunariu, Wei Yuan, Johann S. de Bono

**Affiliations:** 1 The Institute of Cancer Research, 15 Cotswold Road, London SM2 5NG, UK; 2 The Royal Marsden NHS Foundation Trust, Downs Road, Sutton SM2 5PT, UK; 3 GIRT-Uro, Interdisciplinary Group for Translational Research and Clinical Trials, Candiolo Cancer Institute, FPO- IRCCS, SP142, km 3,95, 10060 Candiolo TO, Italy; 4 Institute of Oncology Research, Oncology Institute of Southern Switzerland, Università della Svizzera Italiana, Bellinzona, Switzerland; 5 ETH Zurich, Department of Health Sciences and Technology, Zurich, Switzerland; 6 Department of Medicine, Veneto Institute of Molecular Medicine, Padova, Italy; 7 Department of Pharmaceutical and Pharmacological Sciences, University of Padova, Padova, Italy; 8 Department of Medicine, Columbia University Irving Medical Center, New York, NY 10032, USA; 9 Division of Medical Oncology, Department of Medicine, University of Washington, Fred Hutchinson Cancer Center, Seattle, WA 98109, USA; 10 Janssen Research, Spring House, PA 19477, USA

**Keywords:** B7-H3, Prostate cancer, DNA damage repair, Antibody drug conjugate

## Abstract

**Background:**

B7-H3 is a cell surface immunomodulatory glycoprotein overexpressed in prostate cancers (PC). Understanding its longitudinal expression at emergence of castration-resistance, and association with tumour genomics, is critical to the development of and patient selection for B7-H3 targeted therapies.

**Objective:**

To characterize B7-H3 expression in same-patient, castration-sensitive PC (CSPC) and castration-resistant PC (CRPC) biopsies, associating this with PC genomics, and to evaluate the antitumour activity of an anti-B7-H3 antibody-drug conjugate (ADC) in human CRPC *in vitro* and *in vivo*.

**Design, setting, and participants:**

We performed IHC and next generation sequencing on a cohort of 98 clinically annotated, CRPC biopsies, among which 72 patients had CSPC biopsies for analyses. We analysed two CRPC transcriptome and exome datasets. PC organoids (PDX-Os) were derived from patient-derived xenografts (PDX) generated from human CRPC biopsies.

**Outcome measurements:**

We evaluated B7-H3 mRNA expression in relation to a panel of 770 immune-related genes; compared B7-H3 protein expression between same-patient CSPC and CRPC biopsies; determined associations with PC genomic alterations; evaluated the antitumour activity of DS-7300a, a topoisomerase-1 inhibitor payload anti-B7-H3 ADC, in human PC cell lines, organoids and xenografts of different histologies, B7-H3 expressions, and genomics.

**Results and limitations:**

B7-H3 was one of the most highly expressed immunomodulatory genes in CRPCs. Most CSPCs (97%) and CRPCs (93%) expressed B7-H3 and conversion from B7-H3 positive to negative, or vice versa, during progression from CSPC to CRPC was rare. B7-H3 is overexpressed in tumours with defective DNA repair (DDR) gene (*ATM, BRCA1/2*) alterations compared to tumours without DDR gene alteration. DS7300a had antitumour activity against B7-H3 expressing human PC models including cell lines, PDX-Os and PDXs of adenocarcinoma and neuroendocrine histology.

**Conclusions:**

B7-H3 is overexpressed in the majority of PCs, especially DDR defective tumours. B7-H3 positive CRPC may be targeted with an anti-B7-H3 ADC.

**Patient summary:**

B7-H3, a protein expressed on the surface of most lethal PC, and in particular those with specific mutations, can be targeted using drugs that bind B7-H3 and carry potent chemotherapies. These drugs are being evaluated in cancer patients.

## Introduction

Advanced prostate cancer (PC) is a leading cause of male cancer mortality (1). PC remain largely insensitive to current cancer immunotherapies arguably due to operative potent immunosuppressive mechanisms (2,3). Whilst the immune checkpoint PD-L1 (B7-H1; CD274) is infrequently expressed in PC, another member of the B7 family of immunomodulatory type 1 transmembrane glycoprotein, B7-H3 (CD276), is highly expressed in PC, among other solid tumours, and reported to be associated with worse prognosis (4–7). Overexpressed in placenta, but with relatively low B7-H3 expression in normal tissues, B7-H3 is an attractive therapeutic target (8).

Humans and other primates predominantly express the 4-immunoglobulin (Ig) form of B7-H3, comprising a tandem duplicated pair of immunoglobulin (Ig) variable (V)-like and Ig constant (IgC)-like domain; mice only express the homologous single IgV-IgC protein (9). 4-Ig B7-H3 has been shown to have context-dependent, pleotropic, immunomodulatory functions in autoimmunity, transplantation, and malignancy (10–17). Studies of B7-H3 in both solid tumours and haematological malignancies have demonstrated immune-inhibitory effects mediated by cytotoxic T cells and natural killer cells (10,13,17,18). In contrast, in a study using the TRAMP PC mouse model (RB1 and TP53 loss of function), B7-H3 knockout generated an immunosuppressive tumour microenvironment (TME) and promoted tumour growth, although this model’s predominantly neuroendocrine phenotype differs from most human prostate carcinomas (17). Beyond its immunologic functions, B7-H3 has also been reported to increase tumour survival, stemness, chemo-resistance, and metastases through modulation of pathways such as JAK2-STAT3, MEK, PI3K/AKT, and anti-apoptotic proteins in models of colorectal, breast, ovarian, head and neck, and other cancers (19–21).

Several classes of B7-H3-targeted therapies, including antibody-drug conjugates (ADC), monoclonal and bispecific antibodies, and chimeric antigen receptor (CAR)-T are in (pre)clinical development (22–25), although none are clinically approved to date. ADCs are composed of monoclonal antibodies, joined via a linker, to a biologically active payload allowing for the delivery of potent cytotoxic directly to target-expressing cells, thereby improving therapeutic window (26). Trastuzumab deruxtecan, a HER2-targeting ADC with a cleavable tetra-peptide-based linker and a topoisomerase-1 (TOP1)-inhibitor payload has important antitumour activity against advanced HER2-positive breast and gastric cancer (27,28). Targeting B7-H3 using an ADC with the same linker payload technology may be an effective strategy for treating PC.

The objectives of this study are to describe the longitudinal expression of B7-H3 in same-patient biopsies during progression from castration-sensitive prostate cancer (CSPC) to castration-resistant prostate cancer (CRPC), to associate membranous B7-H3 (mB7-H3) expression with tumour genomics, and to evaluate the antitumour activity of a novel anti-B7-H3 ADC with a TOP1-inhibitor payload (DS-7300a) on human PC cell lines, patient-derived xenograft derived organoids (PDX-Os) and patient-derived xenografts (PDXs) with varying levels of B7-H3 expression, histologies, and genomic backgrounds, in order to support the development of effective B7-H3 targeted therapies and companion biomarkers that improve PC care.

## Materials and Methods

### Patients and tissue samples

Patients with CRPC treated at The Institute of Cancer Research and Royal Marsden Hospital (ICR/RMH) between December 2011 and July 2019 were randomly selected, provided informed consent, and enrolled onto institutional protocols approved by the RMH ethics review committees (reference no. 04/Q0801/60). Eligible patients (n=98) had histologically confirmed formalin-fixed paraffin-embedded (FFPE) CRPC biopsies; 72/98 had diagnostic treatment naïve, CSPC biopsies. Tumour histology was determined by board certified pathologists. Clinical data were retrospectively collected from the hospital medical records (Supplementary Fig. S1, Supplementary Table S1).

### Transcriptome validation cohorts

270 mCRPC transcriptomes generated by the International Stand Up To Cancer/Prostate Cancer Foundation (SU2C/PCF) Prostate Cancer Dream Team (29) were downloaded and reanalysed. An independent cohort of 95 mCRPC transcriptomes from patients treated at RMH/ICR were analysed(30).

### Immunohistochemistry

Immunohistochemistry (IHC) was performed on FFPE tissue sections using an automated staining platform (Bond RX, Leica Biosystems) as described in Supplementary Table S2. Bone biopsies were decalcified using pH 7 EDTA for 48-hours. Antibodies against human B7-H3, ATM, MMR proteins, synaptophysin, chromogranin, CD56, RB1, p21, p16, AR, AR-V7, PTEN, and Ki67 were validated by siRNA knockdown in positive control cell lines, Western blotting, and IHC (Supplementary Methods) (31–34).

### Genomic characterisation and mutation analyses of CRPC biopsies

CRPC biopsies were assessed for defective DNA repair (DDR) gene alterations by Next-generation sequencing (NGS) using previously described methods (35). Patients were defined as having DDR gene alterations, excluding mismatch repair defects (MMRd), based on the gene list in Supplementary Table S3.

### Cell line and PDX-O growth and viability assays

Human PC cells are cultured under conditions described in Supplementary Table S4 and seeded in 96-well plates for drug treatment. PDX-O were derived from PDXs generated from human CRPC biopsies using established methods described in detail in Supplementary Methods(36–38). Briefly, once organoids have formed in Matrigel® matrix (356231, Corning) domes seeded in 24-well plates, they were re-seeded in 96-well plates for drug treatment. PDX-Os and PC cells were treated one day after (re)seeding with DS-7300a (anti-B7-H3 ADC), a non-targeting IgG1-ADC, the parental anti-B7-H3 antibody, the naked cytotoxic payload (deruxtecan [DXd]), or 10 mmol/L acetate buffer-5% sorbitol-pH 5.5 (ABS) vehicle. After 6 days of treatment, the viability of cell lines and PDX-Os were assessed using CellTiter-Glo 2D (G9241, Promega) and CellTiter-Glo 3D (G9681, Promega) viability assays, respectively. All assays were performed with 3 experimental and at least 3 technical replicates.

### PDX studies

PDXs were generated from clinically annotated human CRPC biopsies using previously described methods and characterised by IHC and whole genome sequencing (CP50, CP142) or targeted NGS (CP341, CP327) (36–38) (Supplementary Methods). The original patient tumour biopsies were also characterised by IHC and targeted NGS. Tumour bearing mice were randomised to the following intravenous treatments: DS-7300a (3 mg/kg or 10 mg/kg), non-targeting IgG1-ADC (3 mg/kg or 10 mg/kg), anti-B7-H3 antibody (3 mg/kg or 10 mg/kg), or ABS (3.4 ml/kg) vehicle. Mice (4-8 per arm) were treated when tumours reached ~100 mm^3^, twice, 2-weeks apart. Allocation was concealed from staff administering the drugs, monitoring the mice, and measuring the tumours. Mice were weighed, monitored for distress, and tumours were measured by calliper every 2-3 days. Mice were sacrificed and tumours were collected after 40-days or when tumours reached 1000 mm^3^, whichever occurred first.

### Bioinformatics

Transcriptome data from mCRPC biopsy generated by the SU2C/PCF Prostate Cancer Dream Team (n=270), as well as a separate ICR/RMH validation cohort (n=95), were analysed (3). Transcriptome reads were aligned to the human reference genome (GRCh37/hg19) using TopHat2 (version 2.0.7). Gene expression, as fragments per kilobase of transcript per million mapped reads (FPKM), was calculated using Cufflinks (30).

A publicly available PC single-cell RNASeq (scRNASeq) dataset from 13 patients (12 prostatectomy, 1 lymph node (LN)) was analysed. Raw gene expression count matrices were downloaded from Gene Expression Omnibus (accession: GSM4203181)(39). The R package Seurat (v4.0.3)(40) was used for quality control by removing cells with less than 1000 or more than 4000 distinct genes and more than 5% mitochondrial genes. Cells were log normalized and dimensional reduction was performed prior to clustering. Single cells were annotated with ENCODE cell types using the SingleR (v1.4.1) R package, using the Blueprint ENCODE data, downloaded with the celldex (v1.0.0) R package (41) for reference. Seurat was used for dimensional reduction visualization of cells and gene expression quantification.

### Statistical analysis

All statistical analyses were performed using R or GraphPad Prism version 6 as described in Supplementary Methods.

## Results

### Membranous B7-H3 protein is highly expressed by advanced PC epithelial cells

To validate B7-H3 as a therapeutic target in advanced PC, we analysed two independent PC transcriptome datasets and ranked its expression in comparison to other genes in the 770-genes nCounter PanCancer Immune Profiling Panel (42) and showed that B7-H3 was among the highest expressed immune genes in the SU2C/PCF (6^th^ centile) and RMH cohorts (8^th^ centile). Specifically, expression was significantly higher than that of all other members of the B7 family of immunomodulatory glycoproteins, which includes the immune checkpoints, PD-L1/2 and VISTA (Supplementary Table S5) (42–45).

**Figure 1:**
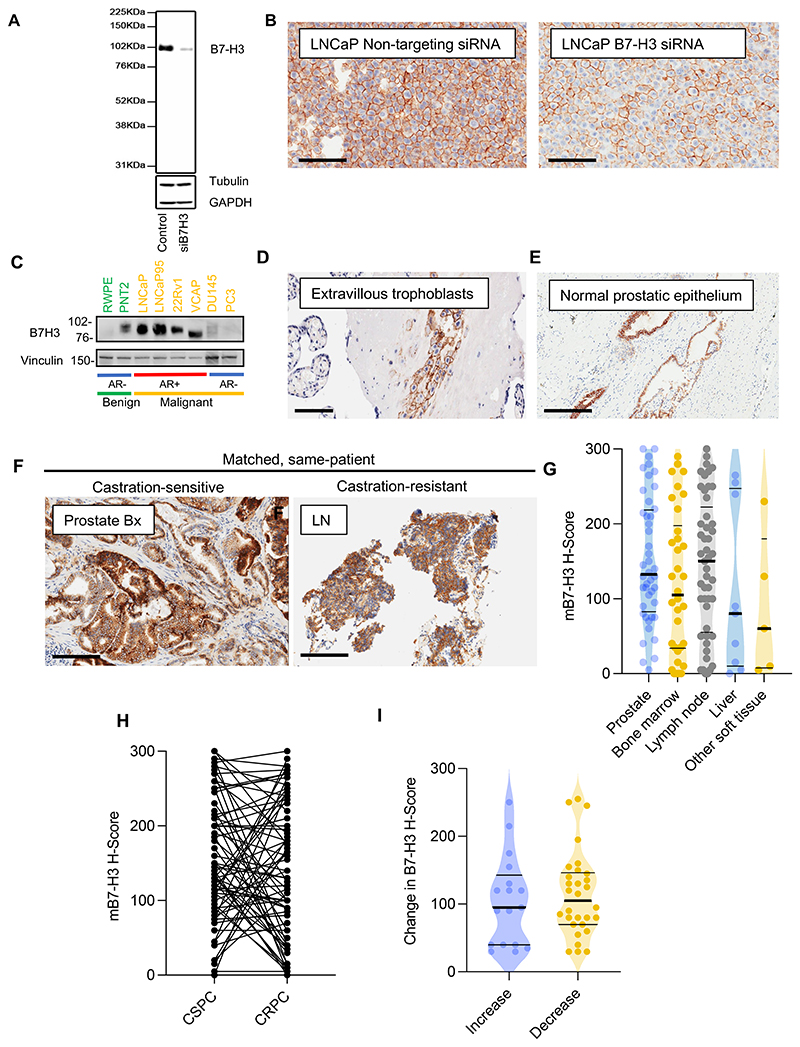
mB7-H3 protein expression in CRPC **(A)** B7-H3 antibody specificity confirmed by Western blotting of whole-cell lysates from LNCaP cell line treated with non-targeting control siRNA and pooled B7-H3 siRNA. **(B)** IHC showing LNCaP cell pellets treated with B7-H3 siRNA or non-targeting control siRNA. **(C)** Western blot showing B7-H3 expression in parental prostate and malignant PC cell lines. **(D, E)** IHC showing B7-H3 staining of extravillous trophoblasts (100 µm scale bar), and normal prostatic epithelium with preferential luminal staining and minimal basal or stromal staining. 200 µm scale bar. **(F)** IHC showing B7-H3 staining in matched CSPC and CRPC biopsies. 200 µm scale bar. **(G)** mB7-H3 expression by biopsy site. Median (IQR) for prostate: 133 (83-219); bone marrow: 105 (34-198); lymph node: 150 (55-223); liver: 249 (10-248); soft tissue: 70 (9-232). **(H)** B7-H3 expression in same-patient CSPC (median: 140, IQR: 91-214) and CRPC (median: 123, IQR: 43-218) biopsies. **(I)** Changes in B7-H3 H-score as tumours progress from CSPC to CRPC. For **(G, H, I)**, horizontal bars denote interquartile ranges and medians. IHC = Immunohistochemistry; CSPC = Castration-sensitive prostate cancer; CRPC = Castration-resistant prostate cancer.

To characterise B7-H3 protein expression, we validated an anti-B7-H3 antibody (clone: D9M2L) against the extracellular IgV1-like domain of B7-H3 conserved across known splice isoforms by showing a reduction in the protein expression of B7-H3 in whole-cell lysates (Western blot) and cell pellets (IHC) from LNCaP cells treated with B7-H3 siRNA (Dharmacon) (9,46) (Fig. 1A, B). B7-H3 was expressed in AR-positive but not AR-negative PC cell lines, and expectedly, by extravillous trophoblasts in the human placenta, and luminal cells of benign prostatic tissue (Fig. 1C-E).

B7-H3 protein expression was evaluated in 98 mCRPC biopsies and 72 same-patient treatment-naïve CSPC biopsies. Twenty-six archival CSPCs were unavailable or of inadequate quality for IHC. Given B7-H3-targeted therapies mostly bind membranous B7-H3 (mB7-H3), we focused on mB7-H3, although we found a strong association between membranous and cytoplasmic B7-H3 expression(n=170, Spearman’s correlation [r_s_] = 0.7; *p*<0.001; Supplementary Fig. S2). mB7-H3 was expressed (defined as H-score [HS] ≥5) by the majority of CRPC (91/98 [93%]) and same-patient, CSPC (70/72 [97%]) biopsies. Median mB7-H3 expression (HS) was 143 (interquartile range [IQR]: 96-214, n=72) in CSPC biopsies and 128 (IQR: 40-223, n=98) in CRPC biopsies. Four CRPC biopsies had features of neuroendocrine differentiation determined IHC staining for synaptophysin, CD56 or chromogranin and/or morphology. These tumours were all B7-H3 positive with HS ranging from 40 to 300.

There was no significant difference in median mB7-H3 HS (Fig. 1H, *p*=0.1) or in the proportion of B7-H3 positive tumours between the 72 same-patient CSPC and CRPC biopsies (*p*=1). mB7-H3 expression changed bidirectionally across same-patient CSPC and CRPC biopsies. In 30 patients, there was a difference in mB7-H3 HS of ≤30 between CSPC and CRPC biopsies. An increase in B7-H3 HS (>30) was observed in 15 patients (median increase: 120, IQR: 40-155) and a decrease in B7-H3 HS (>30) was observed in 27 patients (median decrease: 120: IQR: 80-150) (Fig. H, I). One patient with B7-H3 negative CSPC biopsy had B7-H3 staining on the CRPC biopsy and one patient with mB7-H3 staining in the CSPC biopsies had a mB7-H3 negative CRPC biopsy. There was no significant difference in mB7-H3 expression between the different biopsy sites (*p*=0.2, Fig. 1G).

**Figure 2:**
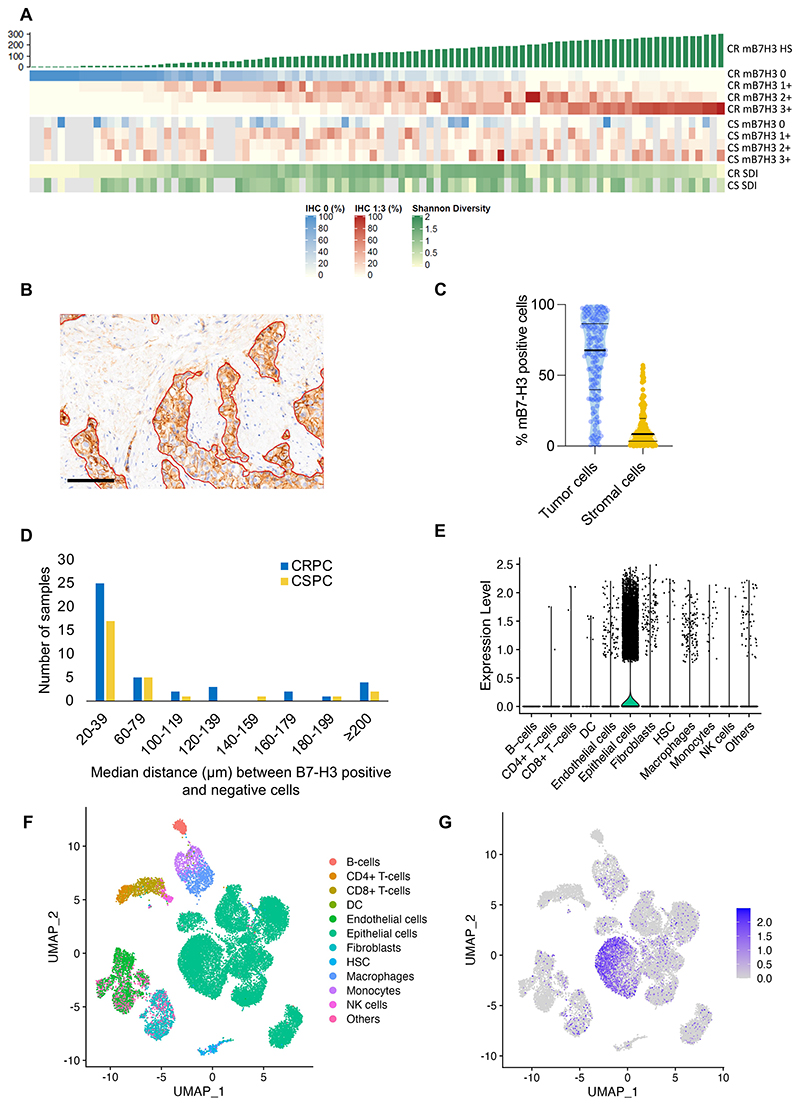
B7-H3 is heterogeneously expressed on CRPC **(A)** Membranous B7-H3 protein expression (HS) and intra-sample heterogeneity quantified by Shannon’s diversity index. **(B)** IHC of mCRPC biopsy with tumour-stroma interface demarcated by red line. 100 µm scale bar. **(C)** Violin plots showing the percentages of B7-H3 positive cells in the tumour and stroma of 98 mCRPC biopsies. B7-H3 was expressed in a significantly higher proportion of tumour cells (median: 67%, IQR: 40-87%) than stromal cells (median: 9%, IQR: 3-20%). Horizontal bars denote IQR and medians. **(D)** Bar graph categorizing CSPC and CRPC samples by the mean distance between mB7-H3 positive and negative tumour cells. **(E, F, G)** scRNASeq data from 13 PC biopsies (12 primary PC; 1 LN metastasis of PC) with clustering by inferred cell type. Corresponding UMAP of B7-H3 expression by inferred cell type clustering **(F)** and relative expression of B7-H3 mRNA by inferred cell type **(G)**. IQR = interquartile range. CSPC = castration-sensitive prostate cancer; mCRPC = metastatic castration resistant prostate cancer; scRNASeq = single-cell RNA sequencing; PC = prostate cancer; HS = Histo-Score. UMAP = Uniform manifold approximation and projection.

We observed intra-sample heterogeneity in mB7-H3 expression, with all B7-H3 positive biopsies also having tumour cells with no B7-H3 expression. Heterogeneity did not differ between CSPC and CRPC (median SDI for CSPC: 0.98; CRPC: 1.06; *p*=0.2) (Fig. 2A). B7-H3 was primarily expressed by tumour and not stromal cells (median tumour cell % positive [IQR]: 68% [40-87%]; stroma: 9% [4-20%]; *p*<0.001) (Fig. 2B, C). mB7-H3-positive tumour cells were mostly within 1-2 cell diameters from mB7-H3 negative tumour cells (median distance [IQR]: 24 *μ*M [16-51 *μ*m]; Fig. 2D). B7-H3 mRNA expression correlated with protein expression (r_s_=0.6, *p*<0.001) so we corroborated this finding using a publicly available scRNASeq dataset from 13 CSPC patients and showed that B7-H3 mRNA was predominantly expressed by tumour cells and not stromal/immune cells. Among stromal cells, B7-H3 mRNA was most commonly expressed by macrophages, fibroblasts, and endothelial cells (Fig. 2E-G).

### mB7-H3 expression and deleterious DNA damage response gene alterations

Given DNA damage and *BRCA2* depletion has been shown to upregulate the B7 family immune checkpoint, PD-L1, and several anti-B7-H3 ADCs in development carry DNA damaging payloads, we evaluated for the association between B7-H3 mRNA expression and DDR gene alterations in the SU2C/PCF (n=270) transcriptome dataset (35,47,48). We showed that expression was indeed higher in samples with DDR gene alterations involved (in)directly in homologous recombination repair than those without (*p=*0.03, DDR gene list in Supplementary Table S3, Supplementary Fig. S3). However, B7-H3 expression was not associated with MMRd. We observed a similar effect in the ICR/RMH transcriptome cohort (n=95, *p*=0.07).

**Figure 3:**
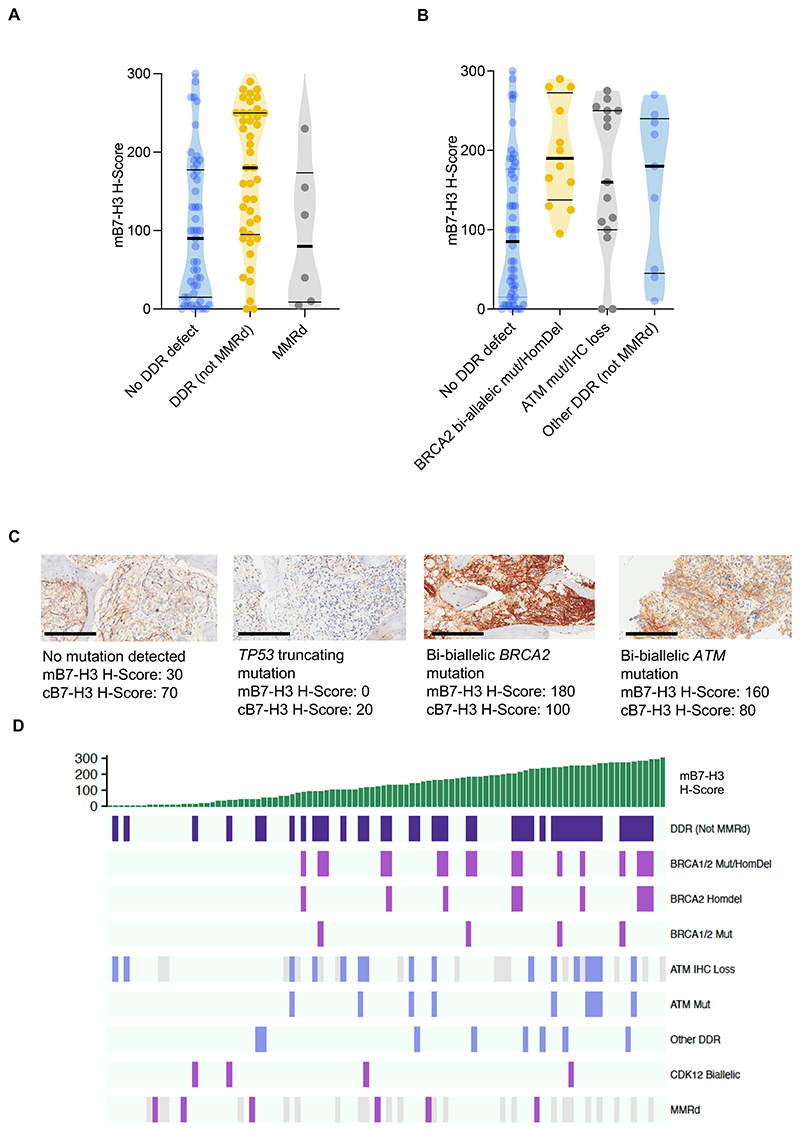
CRPC mB7-H3 expression and deleterious DDR gene alterations **(A)** Violin plots of mB7-H3 expression in tumours without DDR gene alteration (median: 90, IQR: 15-178, n=43), with DDR gene alteration (not MMRd) (median: 180, IQR: 95-250, n=49), and MMRd (median: 80, IQR: 9-174, n=6). B7-H3 was significantly higher in tumours with DDR gene alterations (excluding MMRd) than those without (*p*<0.001). Horizontal bars denote IQRs and medians. *P-*value was calculated using Mann-Whitney U test. **(B)** mB7-H3 expression in tumours without DDR (n=43), *BRCA2* homozygous mutation or deletion (median: 180, IQR: 128-265, n=13), ATM protein loss (median: 150, IQR: 93-250, n=16), other DDR (not MMRd) (median: 200, IQR: 73-243, n=8). B7-H3 expression is significantly higher in tumours with *BRCA2* homozygous mutation or deletion (*p=*0.003) and ATM loss (*p*=0.02) compared with tumours without DDR. Horizontal bars denote IQRs and medians. *P-*values were calculated using Mann-Whitney U test. **(C)** IHC of mCRPC biopsies mB7-H3 expression in tumours with and without DDR gene defects. 200 µm scale bar. **(D)** Profile of DDR gene/protein alteration and pathway alteration in 98 patients with mCRPC. Purple boxes denote the presence of alteration of the specified gene. Light blue boxes denote no detectable alteration of the specified gene. Grey boxes denote cases where ATM or MMR IHC data was not available. IQR = interquartile range; IHC = immunohistochemistry; DDR = DNA damage response; MMRd = mismatch repair defect; HomDel = homozygous deletion; WT = wildtype; Mut = mutation; mB7-H3 = membranous B7-H3; cB7-H3 = cytoplasmic B7-H3.

We then compared mB7-H3 protein expression between mCRPC biopsies and showed that mB7-H3 expression was higher in mCRPC with DDR gene alterations, excluding MMRd (n = 49, median HS [IQR]: 180 [95-250]) than tumours without (n=43; median HS [IQR]: 90 [15-178]; *p*<0.001) (Fig. 3A, Supplementary Table S6). Specifically, higher mB7-H3 was present in tumours with bi-allelic *BRCA2* mutation mutations (n=13, *p=*0.003) or ATM protein loss (n=16, *p=*0.02) than in those without DDR. There was no significant difference in mB7-H3 HS between tumours with MMRd (n=6) and those without DDR (Fig. 3B-D).

### Targeting B7-H3 in patient-derived PC PDX-Os and cell lines

**Figure 4:**
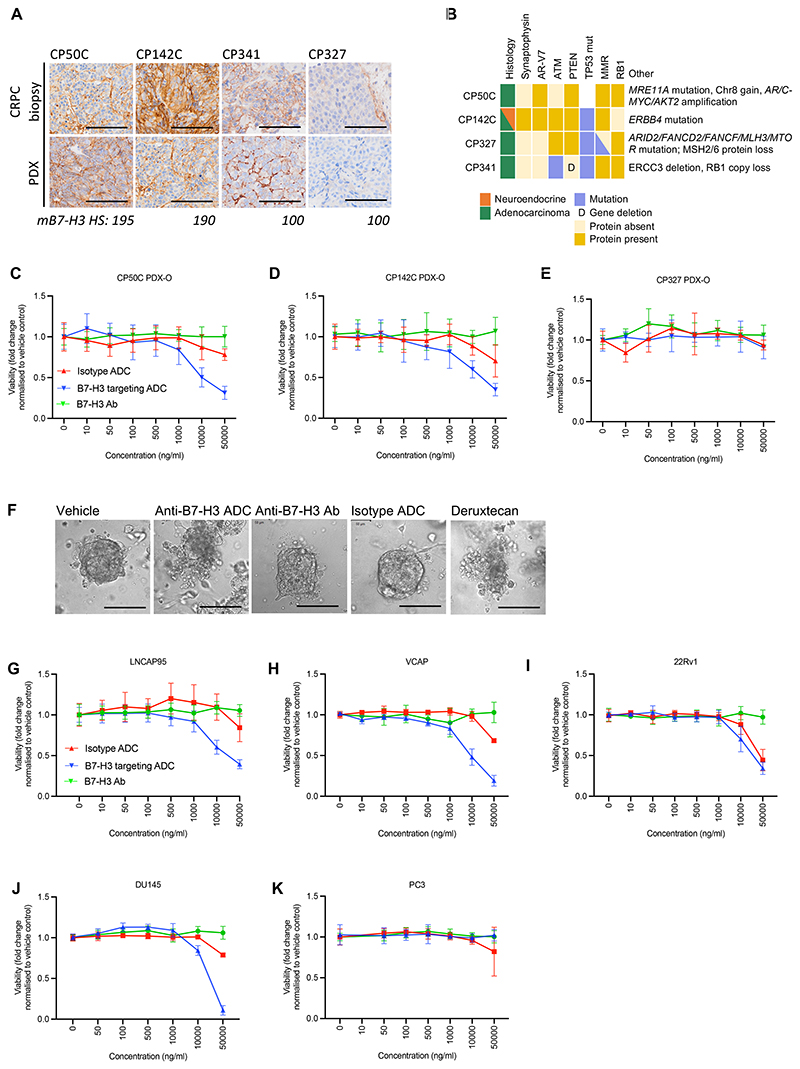
Anti-B7-H3 ADC with a TOP1-inhibitor payload has antitumour activity in human CRPC PC models *in vitro* **(A)** B7-H3 IHC in tumour biopsies and corresponding PDXs used for *in vivo* drug studies and from which PDX-Os were generated for *in vitro* drug studies. 100 µm scale bar. **(B)** Molecular characteristics of PDX models used for B7-H3 drug studies. **(C-E)**. B7-H3 positive (CP50C, CP142C) and B7-H3 negative (CP327) human CRPC PDX-Os were treated with DS-7300a (anti-B7-H3 ADC), parental anti-B7-H3 antibody, non-targeting IgG1-ADC, or DXd. Viability was significantly different between treatment arms for the CP50C (*p*<0.001) and CP142 models (*p*<0.001). Viability was significantly lower in CP50C PDX-Os treated with the anti-B7-H3 ADC compared with those treated with the non-targeting IgG1-ADC at 1000 ng/ml, 10,000 ng/ml, and 50,000 ng/ml (all *p*<0.001). Viability was significantly lower in the CP142C PDX-Os treated with the anti-B7-H3 ADC compared with the non-targeting IgG1-ADC at drug concentrations of 1000 ng/ml, 10,000 ng/ml, and 50,000 ng/ml (all *p*<0.001). **(F)** Confocal imaging showing CP50C PDX-Os after 6 days of treatment with the anti-B7-H3 ADC, parental anti-B7-H3 antibody, non-targeting IgG1-ADC, or DXd payload. 100 µm scale bar. **(G-K)** B7-H3 positive and B7-H3 negative PC cell lines were treated with DS-7300a (anti-B7-H3 ADC), parental anti-B7-H3 antibody, non-targeting IgG1-ADC, DXd payload, or vehicle. Significant differences in (*p*<0.001) in cell viability were observed between the different treatment arms in LNCaP95 (500 ng/ml, 1000 ng/ml, 10,000 ng/ml and 50,000 ng/ml), VCAP (500 ng/ml, 1000 ng/ml, 10,000 ng/ml and 50,000 ng/ml), 22RV1 (10,000 ng/ml and 50,000 ng/ml), and DU145 (10,000 ng/ml and 50,000 ng/ml) prostate cancer cell lines. Cell viability was significantly lower in cells treated with anti-B7-H3 ADC compared with the non-targeting IgG1-ADC at these concentrations in LNCaP, VCAP, and DU145 cells (all *p*<0.001). Cell viability was significantly lower in cells treated with anti-B7-H3 ADC compared with the parental anti-B7-H3 antibody at these concentrations in LNCaP, VCAP, 22RV1, and DU145 cells (all *p*<0.001). For all viability experiments **(C-E, G-K)**, mean viability and standard deviation from 3 individual experiments with at least 3 technical replicates per experiment are shown. Viability was determined using CellTiter-Glo 2D viability assay after 6 days. *P*-values for comparisons across all three treatment groups were calculated by one-way ANOVA and *p-*values for comparisons between two treatment arms were calculated using unpaired Student’s t-test.

Next, we investigated the antitumour activity of a TOP1 inhibitor payload anti-B7H3 ADC (DS-7300a, Daiichi Sankyo) (24) in B7-H3-positive and negative human PC cell lines and PDX-Os of adenocarcinoma (PDX-O: CP327, CP50C; cell line: VCAP, LNCaP, 22Rv1) and neuroendocrine histology (PDX-O: CP142C; cell line: DU145, PC3) with various genomic backgrounds (Fig. 4A, B, Supplementary Table S7)(36,37). All patients from whom the PDX-Os were derived had progressed on standard-of-care PC treatments including taxane chemotherapy and next generation anti-androgens (Supplementary Fig. S4). Of note, both CP50C and CP142C are B7-H3 positive and express AR-V7. CP50C also has ATM protein loss and amplification of *AR, AKT*, and *C-MYC*. CP142C harbours deleterious *ERBB4* and *TP53* mutations and is synaptophysin-positive by IHC. The CP327 model does not express B7-H3 and has MMRd and mutations of *TP53, FANCD2, FANCF, ARID2*, and *MTOR*. In comparison with the vehicle control, parental anti-B7-H3 antibody, and non-targeting IgG1-ADC, the anti-B7-H3 ADC (DS-7300a) demonstrated significant antitumour activity in the B7-H3-positive cell lines (VCAP, LNCaP95, 22Rv1, DU145) and PDX-Os derived from tumours with adenocarcinoma (CP50C) and adenocarcinoma with neuroendocrine differentiation (CP142C) at DS-7300a concentrations of 1000 ng/ml or higher. There was no clear association between degree of B7-H3 positivity and tumour shrinkage, although no antitumour activity was observed in the models where B7-H3 was largely absent (PC3 and CP327C) despite these being more payload sensitive (Fig. 4C-K, Supplementary Fig. S5). In LNCaP95, VCAP cell lines, and CP142 PDX-Os, significant antitumour activity compared with the vehicle control and non-targeting IgG1-ADC was also observed at DS-7300a concentrations of 500 ng/ml. The CP142 PDX-O has similar B7-H3 expression to CP50C but is more sensitive to payload (Supplementary Fig. S5B). Whilst the non-targeting IgG1-ADC exhibited some non-specific antitumour activity at the highest concentration (50,000 ng/ml), this was comparably less than that of DS-7300a, except for in 22Rv1 where tumour cell kill by the IgG-1ADC was observed at 10,000 ng/ml.

### Targeting B7-H3 in mCRPC patient-derived xenografts

**Figure 5:**
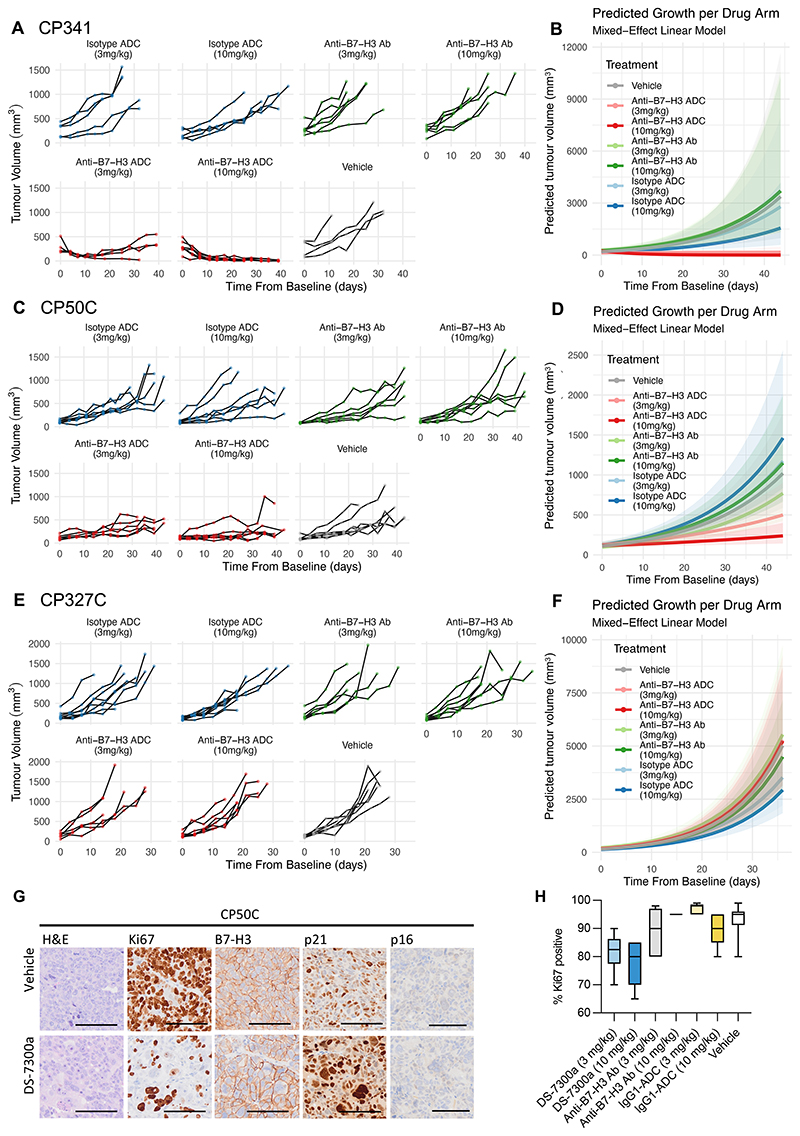
Anti-B7-H3 ADC with a TOP1-inhibitor payload has antitumour activity in human CRPC PC models *in vivo*. **(A, C, F)** Individual tumour volumes of CP341, CP50C, and CP327 PDX models over time in PDXs treated with DS-7300a (anti-B7-H3 ADC), parental anti-B7-H3 antibody, non-targeting IgG1-ADC, or vehicle (3.4 ml/kg). **(B**,**D**,**F)** Predicted tumour growth using a linear mixed effects model of CP341, CP50C and CP327 PDXs treated with DS-7300a (anti-B7-H3 ADC), anti-B7-H3 antibody, non-targeting IgG1-ADC, or ABS vehicle. **(G)** Representative IHC of end-of-treatment CP50C tumours from mice treated with vehicle control or DS-7300a. 100 µm scale bar. **(H)** Ki67 expression (% positive cells) in end-of-treatment CP50C tumours. Tumour Ki67 was significantly lower in mice treated with the anti-B7-H3 ADC (10 mg/kg) than those treated with the non-targeting IgG1-ADC (10 mg/kg, *p*<0.001), the anti-B7-H3 antibody (10 mg/kg, *p*<0.001), and vehicle (*p*<0.001). Tumour Ki67 in mice treated with DS-7300a (3 mg/kg) was significantly lower than those treated with the non-targeting IgG1-ADC (3 mg/kg, *p<*0.001) and vehicle (*p*<0.001). Bars represent maximum and minimums, interquartile ranges, and median. *P-*values were calculated using the unpaired Student’s t-test.

We then evaluated the *in vivo* antitumour activity of DS-7300a, the non-targeting IgG1-ADC, and parental anti-B7-H3 antibody at 3 mg/kg and 10 mg/kg, administered twice, two-weeks apart, in three human mCRPC PDXs (CP341, CP50C and CP327) with varying B7-H3 expression and genomic characteristics (Fig. 4A, B, Supplementary Table Fig. S4, Supplementary Table S7). DS-7300a demonstrated potent, dose-dependent antitumour activity compared with vehicle, the anti-B7-H3 antibody, and the non-targeting IgG1-ADC in both B7-H3-positive PDXs (CP341, CP50C) but not in the B7-H3-negative PDX (CP327C) despite it being more payload sensitive when grown as PDX-Os *in vitro* (Supplementary Fig. S5, Supplementary Table S7). Growth retardation was observed in CP50C PDXs. In CP341 PDXs, we observed tumour regression, including several cases of complete tumour responses at the 10 mg/kg dose (Fig. 5A-F, Supplementary Fig. S6). We did not observe associations between mB7-H3 HS and response to DS-7300 among the B7-H3 positive tumours. Although mB7-H3 expression was lower in CP341 PDXs than the ATM-loss CP50C PDX, the CP341 CRPC tumour also harboured pathogenic *ATM* and *TP53* mutations as well as *RB1* alteration and *ERCC3* deletion (Fig. 4A, B). The patient from which CP341 tumours were derived subsequently had partial radiologic and biochemical response to epirubicin, carboplatin and 5-fluorouracil suggesting the presence of replication stress. Mice did not lose weight or become moribund in the context of any treatment.

Pharmacodynamic analyses of end-of-treatment CP50C and CP341 tumours confirmed the antiproliferative effect of DS-7300a shown by a marked reduction in Ki67 in PDX-Os treated with DS-7300a (10 mg/kg) compared with the vehicle, non-targeting IgG1-ADC, and anti-B7-H3 antibody at corresponding concentrations. In both models, DS-7300a-treated PDX tumours exhibited morphologic features of senescence, characterised by the flattening and enlargement of the cells, irregular and enlarged nuclei, and vacuolization. This was associated with increases in the intensity of expression of the senescence marker, p21. Neither model expressed p16, required in some cells for senescence maintenance. Interestingly, in both responding models, post-treatment tumour B7-H3 expression was similar to that of the parental PDX tumours and did not differ across the treatment arms indicating the presence of potential B7-H3-expression independent resistance mechanisms (Fig. 5G-H, Supplementary Fig. S7).

## Discussions

B7-H3 is one of the most highly expressed immunomodulatory molecules in PC, with numerous B7-H3 targeted therapies currently in clinical development. To our knowledge, this is the first study to characterize B7-H3 protein expression during progression from CSPC to CRPC in matched, same-patient biopsies and to identify the association between B7-H3 expression and DDR gene alterations in PC. In light of this, we evaluated the antitumour activity of DS-7300a, an anti-B7-H3 ADC with a TOP1 inhibitor (DXd) payload, and demonstrated B7-H3 dependent antitumour activity in human advanced PC models *in vitro* and *in vivo*.

We showed that B7-H3 is highly expressed in a significant subset of patients with metastatic CRPC, including those with neuroendocrine differentiation. Whilst B7-H3 overexpression has previously been associated with metastases and worse outcomes (4–7), we observed no significant difference in the rate of B7-H3 positivity or the median level of expression between CSPC and CRPC in our cohort of patients who all developed mCRPC, suggesting that B7-H3 upregulation likely occurred early in the disease course of aggressive tumours. Further, rarely was there a switch from tumours being B7-H3 positive CSPC to B7-H3 negative CRPC, or vice versa. This is relevant for biomarker development for B7-H3 targeting since if the threshold of target expression necessary for drug response is low, biomarker analyses could be performed on archival tissue, and patients may be spared of the need for invasive, fresh CRPC tumour biopsies. Whilst we observed intratumoural heterogeneity in B7-H3 expression, the close proximity between B7-H3 positive and negative cells bears relevance for the clinical development of anti-B7-H3 ADCs – particular those with permeable payloads like DS-7300a – that could engage in bystander kill (26,49).

We orthogonally demonstrated using independent CRPC cohorts that B7-H3 expression associated with homologous recombination repair defects (HRD) but not MMRd, suggesting a link between expression and DNA double strand breaks and repair. Cancers with genomic instability induced by microsatellite instability, HRD, chemotherapy or radiation have been reported to upregulate other B7 family members, such as PD-L1, through JAK-STAT-IRF1 signalling and sensitise to PD-L1 targeted therapies (47,50,51). Whilst PD-L1 is expressed in less than a quarter of PC (7,52), B7-H3 overexpression specifically in HRD tumours is in keeping with the evidence that tumours with DDR can activate specific immune evasive mechanisms (31,53), and supports further mechanistic studies of B7-H3 modulation and rational combination therapeutics targeting B7-H3 in these PC genomic subsets.

Given B7-H3 is often overexpressed in mCRPC, particularly those with HRD, we evaluated the antitumour activity of DS-7300a, a TOP1-inhibitor payload anti-B7-H3 ADC in tumours with varying levels of B7-H3 expression and genomic backgrounds including HRD. We showed B7-H3-dependent, dose-dependent, cytotoxicity *in vitro* and *in vivo* across tumours with both neuroendocrine differentiation and adenocarcinomas. Consistent with clinical studies of other DXd-payload ADCs, we showed that target expression was necessary for response, but additional factors clearly impacted this (54,55) as tumour cells remaining after DS-7300a treatment in the two B7-H3 positive PDX models still expressed levels of B7-H3 comparable to that of the parental tumour and control drug treated tumours.

Response to ADCs depends on the complex interplay multiple factors including target expression and turnover, ADC stability, drug-stromal interactions, drug internalisation, processing, degradation, efflux, tumour genomic background and payload sensitivity(26). Interestingly, CP341 (*RB1* alteration, *ATM/ERCC3/TP53* mutant, adenocarcinoma) PDX-Os were more sensitive to DS-7300a than CP50C (*ATM* loss, adenocarcinoma) despite having lower target expression. TOP1 inhibitors cause replication stress and tumour cell kill by trapping topoisomerase cleavage complexes, which leads to blocking of DNA re-ligation, generation of single-, and subsequently double-strand breaks(56). The combination of deleterious alterations of *ATM*, involved in sensing DNA damage and initiating DDR, *RB1*, involved in DSB repair by canonical non-homologous end joining, and *ERCC3*, involved in nucleotide excision repair, have been shown to confer synthetic lethality with and sensitise to TOP1 inhibition (56–58).

*In vivo* tumour models which responded to DS-7300a showed morphologic features of senescence, downregulated Ki67, and upregulated p21, a marker of senescence. P16 has been shown to be upregulated at later stages of senescence and is important for the maintenance of senescence after cell cycle arrest is first triggered by p21 signalling in fibroblast models, therefore these PC models’ loss of p16 protein led us to posit senescence reversal as a potential resistance mechanism(59). Overall, our results indicate that whilst B7-H3 expression is necessary for response, the level of expression did not correlate with response, and therefore patient selection for B7-H3-targeting needs to consider additional factors such as alterations of DDR genes, *RB1, TP53*, cell cycle and senescence regulation. Elucidating these factors may also guide dose-finding decisions during ADC development that can be personalised for specific PC subsets.

Limitations of this study are as follows. We showed an association between B7-H3 expression and DDR gene defects, but causality and mechanisms through which DDR impacts B7-H3 expression is beyond the scope of this report and being evaluated. CSPC and CRPC biopsies were taken from a single time point from one disease site, therefore do not address potential intra-patient, inter-metastases heterogeneity, or changes in expression in response to treatment; these are best addressed through serial imaging and autopsy studies. Whilst DS-7300a was tolerated by PDXs, the drug does not bind murine 2-Ig B7-H3, therefore on-target, off-tumour toxicity need to be studied in primates (24). The immunologic impact of DS-7300a are beyond the scope of the current study since the PDXs are largely immunodeficient. Finally, analyses of potential markers of response to DS-7300a are hypothesis-generating and requiring interrogation in adequately powered, prospective, clinical cohorts.

## Conclusions

In summary, we show that B7-H3, a druggable member of the B7 family of immune-modulatory glycoproteins, is associated with DDR gene defects, including deleterious *BRCA2* and *ATM* alterations in PC. B7-H3 targeting using an anti-B7-H3 ADC with a TOP1-inhibitor payload has antitumour activity in PC *in vitro* and *in vivo* expressing B7-H3. Molecular characteristics impacting DDR and replication stress and need to be interrogated as part of a biomarker suite for this therapy. Clinical trials evaluating DS-7300a in solid tumours are ongoing (NCT04145622, NCT05280470).

## Supplementary Material

Supplementary Figures

Supplementary Methods

Supplementary Tables

Supplementary Figures Legends
