# Supplementary material for: B7-H3 as a therapeutic target in advanced prostate cancer
Source: Eur Urol. Author manuscript; Available in PMC 2025 Aug 3. (PMC7617982; doi:10.1016/j.eururo.2022.09.004)

Supplementary Figures

Supplementary Figure S1

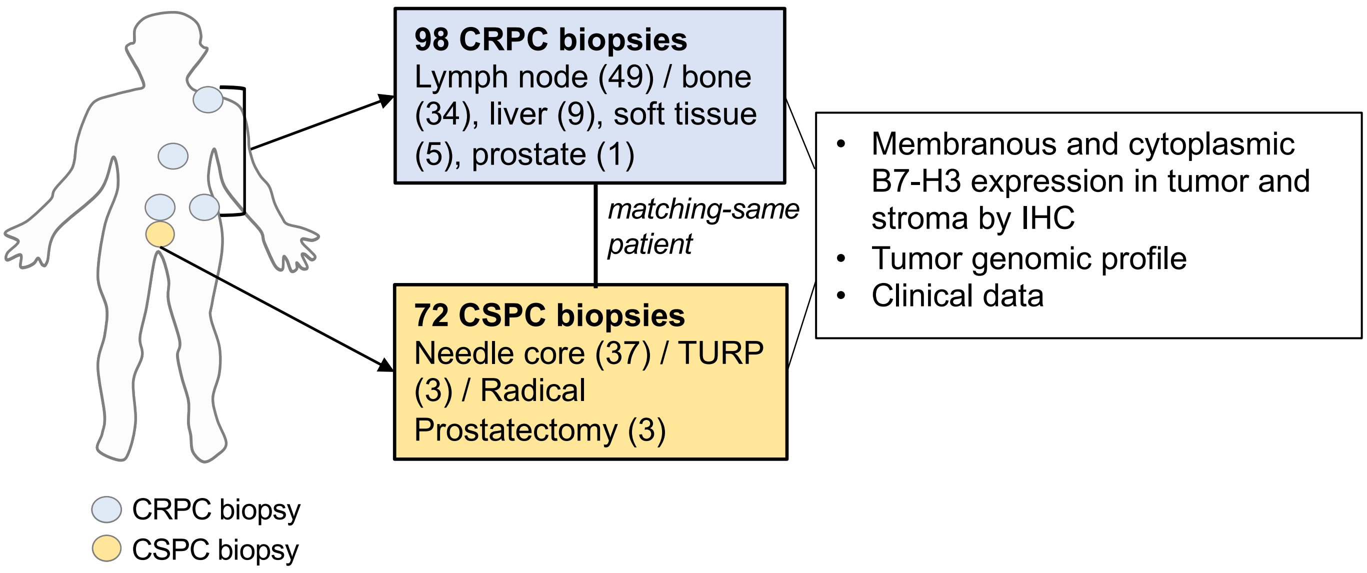

Supplementary Figure S2

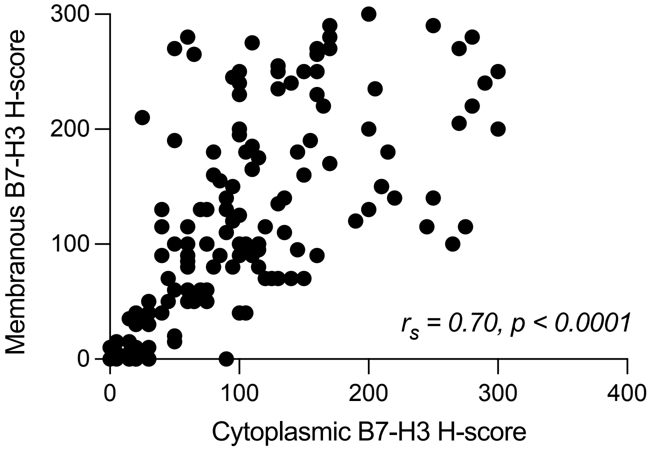

Supplementary Figure S3

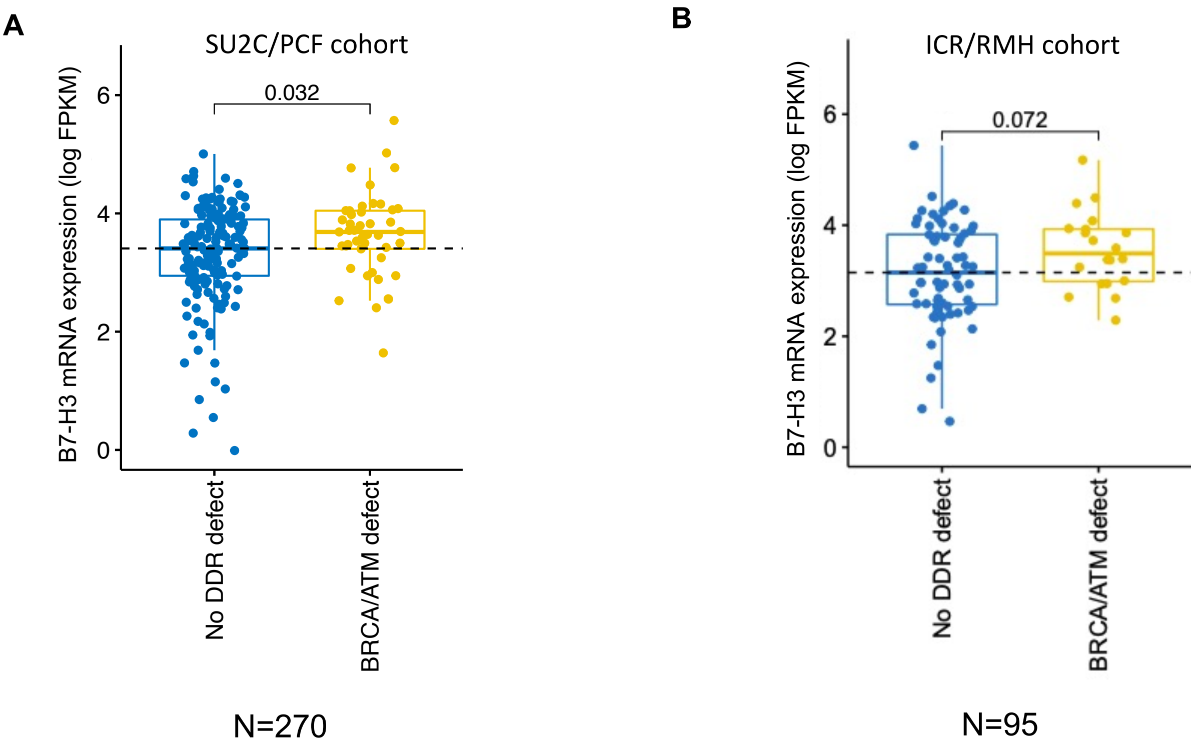

Supplementary Figure S4

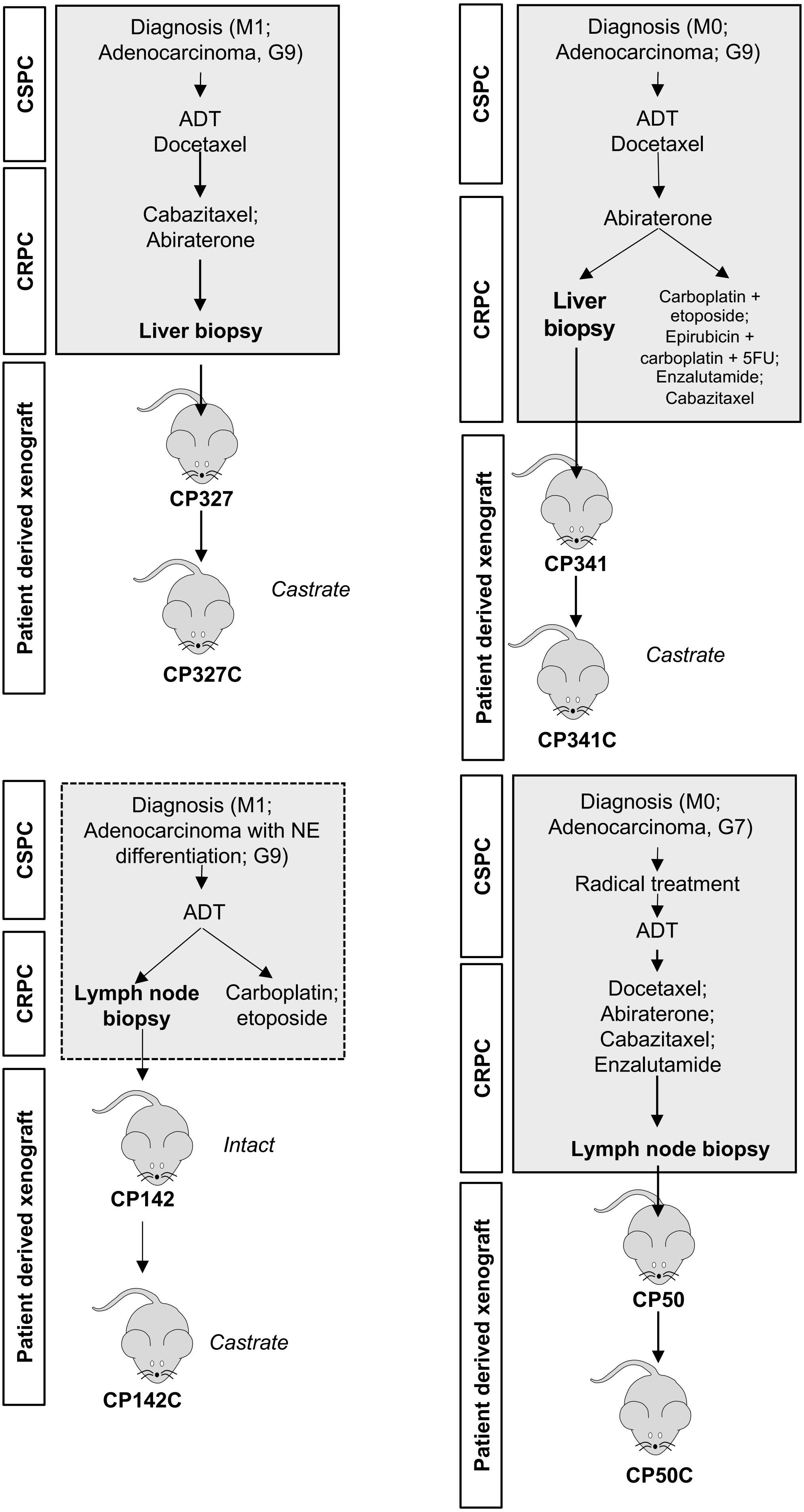

Supplementary Figure S5

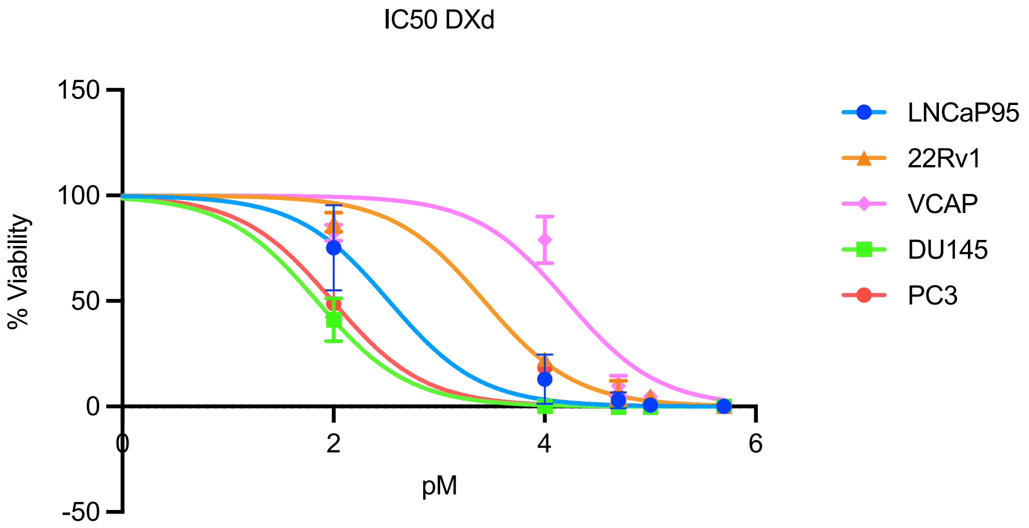

Model	LNCaP95	22RV1	VCAP	DU145	PC3
IC50 (nM)	0.33	2.67	16.23	0.07	0.10

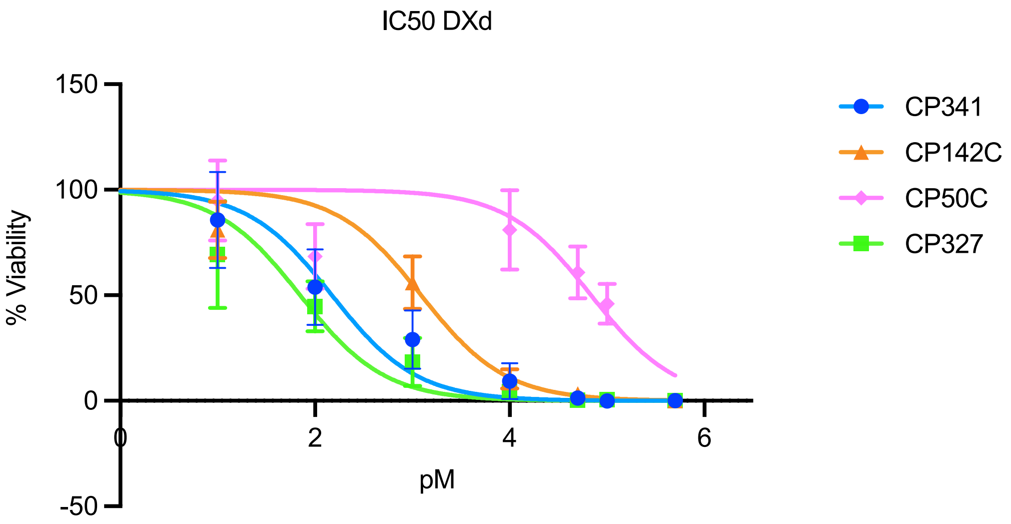

Model	CP341	CP327	CP142C	CP50C
IC50 (nM)	0.12	0.07	1.23	68.51

Supplementary Figure S6

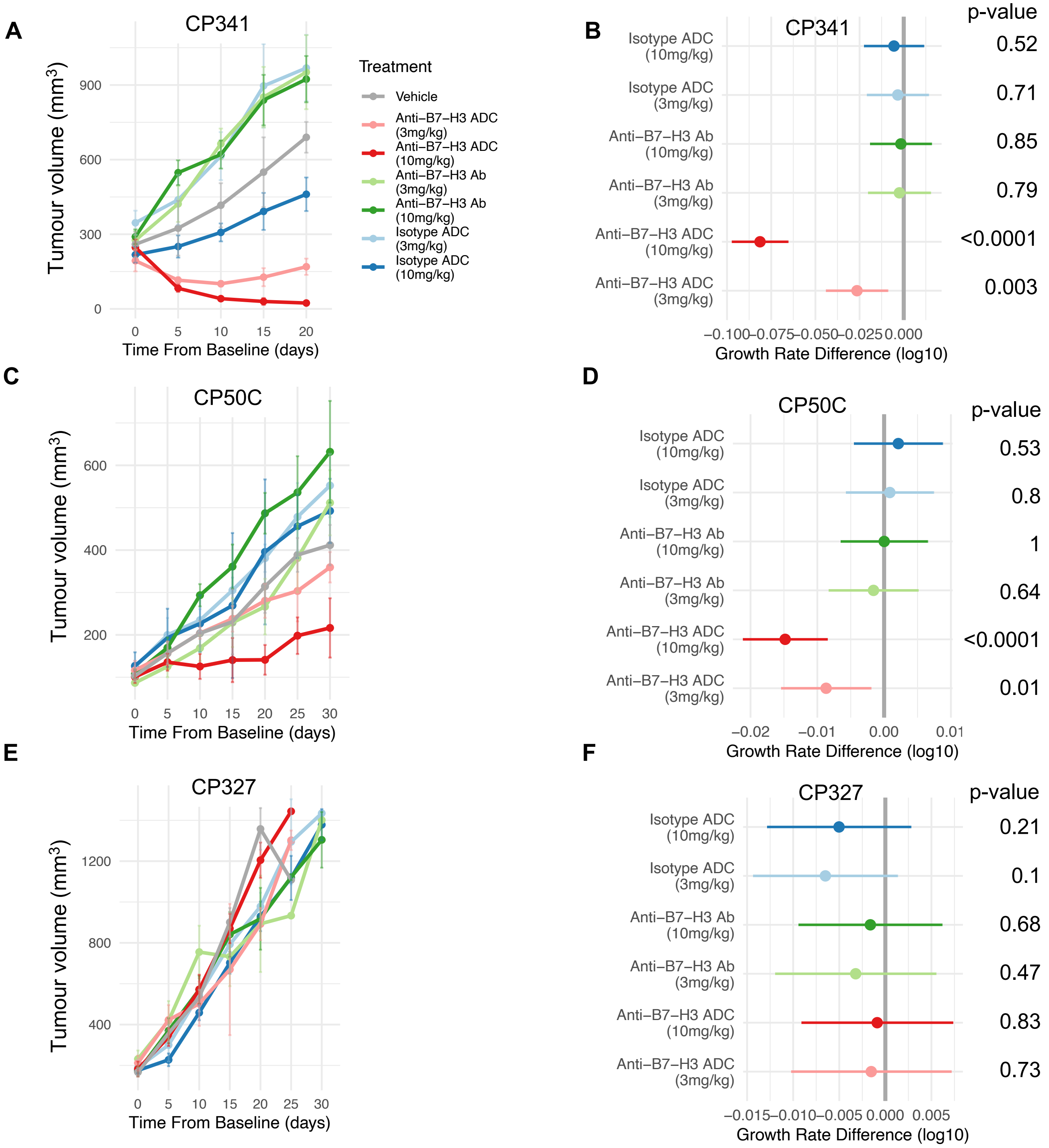

Supplementary Figure S7

A

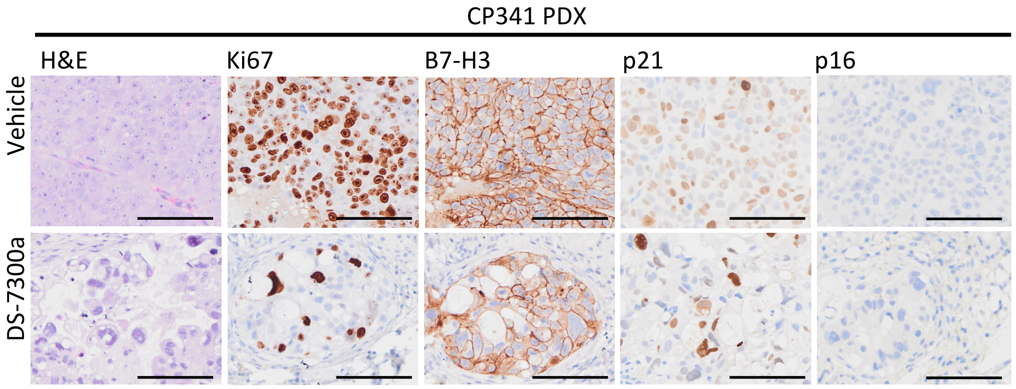

B

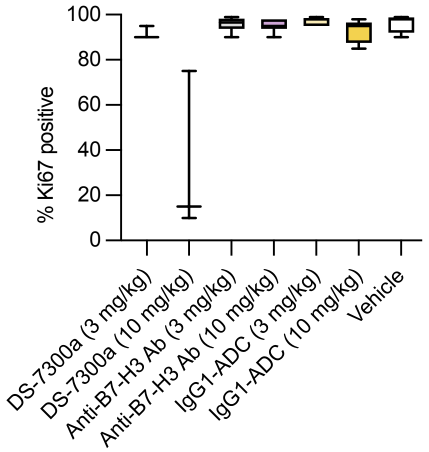

Supplement: Supplementary Figures [file EMS207305-supplement-Supplementary_Figures.pdf]
