# Supplementary material for: B7-H3 as a therapeutic target in advanced prostate cancer
Source: Eur Urol. Author manuscript; Available in PMC 2025 Aug 3. (PMC7617982; doi:10.1016/j.eururo.2022.09.004)
Supplement: Supplementary Methods [file EMS207305-supplement-Supplementary_Methods.docx]

**SUPPLEMENTARY DATA**

***Supplementary Materials and Methods***

## *Antibody validation and IHC*

Antibodies against B7-H3, MMR proteins, ATM, PTEN, RB1, TP53, AR, AR-V7, synaptophysin, chromogranin, CD56, Ki-67, p16 and p21 were validated by Western blot comparing detection of protein expression in whole cell lysates cultured with either nontargeting control siRNA or ON-TARGETplus pooled siRNA against the target protein (Dharmacon). Validation for MMR proteins, ATM, PTEN, RB1, TP53, AR, AR-V7, synaptophysin, chromogranin, CD56, and Ki-67 were as previously described (1-5). Staining conditions for these markers are summarised in Supplementary Table S5. Bone biopsies were decalcified using pH 7 EDTA for 48 hours at 37^o^C.

B7-H3 IHC was performed using a rabbit anti-human B7-H3 monoclonal antibody (#14058, Cell Signalling Technology) that was generated by immunizing animals with a synthetic peptide corresponding to residues surrounding alanine 94 of human B7-H3. Antigen retrieval was achieved by microwaving slides in pH 8.1 Tris-EDTA antigen retrieval buffer for 18-minutes at 800W prior to incubation with an anti-B7-H3 antibody (1:100 dilution) for 1-hour at room temperature on the BioGenix i6000 autostainer (Launch Diagnostics). The reaction was visualized using the EnVision system (K4061; Dako, Agilent Technologies). LNCaP non-silencing siRNA, LNCaP B7-H3 siRNA pellets and normal prostate were used as positive controls and rabbit IgG as negative control.

All the sections were counterstained with hematoxylin. Tissue segmentation was achieved by a supervised machine learning algorithm to recognize prostate cancer foci and surrounding stroma (Halo^TM^, Indica Labs). Cell segmentation was achieved with nuclear DAPI counterstain. Membranous (mB7-H3) and cytoplasmic B7-H3 (cB7-H3) expression for each sample was semi-quantitatively assessed by a pathologist blinded to clinical and molecular data using modified H-scores (HS) (% of weak staining $\times$ 1] + [% of moderate staining $\times$ 2] + [% of strong staining $\times$ 3]) to determine the overall percentage of B7-H3 positivity across the entire stained tumour sample, yielding a range from 0 to 300 (6). The degree of heterogeneity in B7-H3 expression was quantified using the Shannon Diversity Index. MMR and ATM IHC were performed and analyzed using previously described methods (1-4). ATM loss was defined as a nuclear HS of 0. Scoring of the MMR proteins, MSH2, MSH6, MLH1, and PMS2, was achieved by segregating cases in a binary fashion between positive and negative using College of American Pathologists Criteria for biomarker reporting in colorectal carcinoma (7). Percentage of positive cells was determined for Ki-67. Nuclear and cytoplasmic expression was assessed for p16 and nuclear for p21 using modified HS as described above.

***Development of lethal prostate cancer PDX (CP341, CP327)***

The CP341 and CP327 PDXs models were developed as previously described for CP50 and CP142(5,8,9). Briefly, metastatic liver biopsies (one for CP341 model and one for CP327 model) from separate patients with CRPC were divided and implanted subcutaneously into an intact non-obese diabetic (NOD) scid gamma (NSG) (NOD.Cg-Prkdcscid Il2rgtm1WjI/SzJ) male mice between 7–8 weeks. Tumour growth was observed 6 months after implantation. Passaging of tumours was performed by implanting tumour fragments of 3x3x3 mm^3^ subcutaneously into male NSG mice. All experimental protocols were monitored and approved by The Institute of Cancer Research Animal Welfare and Ethical Review Body, in compliance with guidelines specified by the UK Home Office Animals (Scientific Procedures) Act 1986 and the United Kingdom National Cancer Research Institute guidelines for the welfare of animals in cancer research (10).

***PDX-O generation***

Tissue was mechanically dissociated into small pieces in Petri dishes using sterile scalpel blades mixed with sterile PBS and additional ROCK inhibitor Y27632 at 10 µM (S1049, Selleckchem). Sample was filtered through cell strainers 40µm (431750, Corning). Red blood cell lysis was performed using ammonium chloride solution (07850, StemCell Technologies). If a large piece of tissue remained undissociated, cell strainers were used to obtain a pellet for single cell suspension. Samples were rinsed with cold PBS-containing Y27632, centrifuged at 2000rpm for 5 minutes and CellTrics® 50µm (Sysmex) was used to generate single cell suspensions. Cells were embedded at a ratio of 2/1 (matrix:cells) onto Matrigel® (356231, Corning). A standard sterile 24-well plate was pre-warmed at 37oC before use, an aliquot of 50 μl/well was placed in the center of the well; the plate was turned upside down to generate a ‘hanging-drop’ and immediately incubated at 37°C. Freshly prepped culture medium was overlaid and replenished weekly. PDO cultures were monitored daily and organoid visualized after 3-5 days. PDO culture largely followed the protocol published by Drost et al., 2016 using epithelial prostate niche factors: EGF, Noggin and R-Spondin (Peprotech) (11). Organoid growth conditions were optimized for each PDX-O model. PDO base media was modified with additional metabolites and growth factors to improve proliferation and viability. Additional factors included: Glutamine (Gibco), Sodium Pyruvate (Sigma), and recombinant human NRG1 (100-03, Peprotech).

***PDX-O growth***

PDOs were embedded in Matrigel® matrix (356231, Corning) and seeded in replicates in 96-well format optical plates (3904, Corning). Luminescence was detected using CellTiter-Glo® 3D (G9681, Promega) according to manufacturer’s protocol and readings were performed using Gen5 microplate reader (Biotek). Measurements started when organoids developed (baseline, day 0) and proliferation/viability was assessed after 6-days.

***Cell line culture***

All cell lines used in this study were sourced from suppliers and grown in recommended media described in Supplementary Table S4. Cells were cultured at 37°C in 5% CO2 and were tested for mycoplasma using the VenorGem One Step PCR Kit (Cambio) every 3 months and STR-profiled using the Cell authentication service by Eurofins Medigenomix.

***Statistical analyses***

Immunomodulatory molecule mRNA and protein expression was presented using descriptive statistics. Spearman’s rank-order correlations determined the association between B7-H3 mRNA and protein expression. Differences in the proportion of tumours expressing mB7-H3, and mB7-H3 HS, between paired CSPC and CRPC samples, were compared by Fisher’s Exact test and McNemar’s test, respectively. Differences in the % of tumour and stromal cells expressing mB7-H3 in the tumour and stroma component of the same biopsy were compared using McNemar’s test. Differences in B7-H3 protein and mRNA between tumours with different DNA damage response (DDR) gene alterations, and tumours without DDR gene alterations, were compared with the Mann-Whitney U test. Differences in B7-H3 protein expression across disease sites were compared with Kruskal-Wallis test. Shannon Diversity Index between paired CSPC and CRPC samples was compared using Wilcoxon signed-rank test. Linear regressions for associations in transcriptome data were performed. For cell viability assays, mean growth (normalized to vehicle) with standard deviation from four individual experiments were presented. One-way ANOVA was used to calculate *p-*values when comparing cell viability across treatment arms for each cell line and PDX-O model. Post-hoc comparisons of viability between two treatment groups of interest (i.e. DS-7300a vs. the non-targeting IgG1-ADC, DS-7300a vs. the parental anti-B7-H3 antibody) were then calculated using the unpaired Student’s t-test. For PDX tumour growth experiments, tumour volume over time was modelled longitudinal mixed effects model. The model included treatment arm and time (in days from baseline) as well as their interaction as fixed effects and per-mouse random intercept and slope. Estimates and *P-*values of interest refer to the interaction term of treatment arm and time, the difference in tumour volume growth (per day) between each treatment arm and the vehicle arm. No adjustment for multiple testing has been performed. All analyses were conducted using R 4.1.1 or GraphPad Prism v6. The linear mixed effects model was performed using the R package nlme.​

**References**

1. Rodrigues DN, Rescigno P, Liu D, Yuan W, Carreira S, Lambros MB*, et al.* Immunogenomic analyses associate immunological alterations with mismatch repair defects in prostate cancer. *J Clin Invest* **2018**;128(11):5185 doi 10.1172/JCI125184.

2. Ferraldeschi R, Nava Rodrigues D, Riisnaes R, Miranda S, Figueiredo I, Rescigno P*, et al.* PTEN protein loss and clinical outcome from castration-resistant prostate cancer treated with abiraterone acetate. *Eur Urol* **2015**;67(4):795-802 doi 10.1016/j.eururo.2014.10.027.

3. Sundar R, Miranda S, Rodrigues DN, Chénard-Poirier M, Dolling D, Clarke M*, et al.* Ataxia Telangiectasia Mutated Protein Loss and Benefit From Oxaliplatin-based Chemotherapy in Colorectal Cancer. *Clinical Colorectal Cancer* **2018**;17(4):280-4 doi <https://doi.org/10.1016/j.clcc.2018.05.011>.

4. Rescigno P, Gurel B, Pereira R, Crespo M, Rekowski J, Rediti M*, et al.* Characterizing CDK12-Mutated Prostate Cancers. *Clinical Cancer Research* **2020** doi 10.1158/1078-0432.Ccr-20-2371.

5. Gil V, Miranda S, Riisnaes R, Gurel B, D'Ambrosio M, Vasciaveo A*, et al.* HER3 Is an Actionable Target in Advanced Prostate Cancer. *Cancer Research* **2021**;81(24):6207-18 doi 10.1158/0008-5472.Can-21-3360.

6. Detre S, Saclani Jotti G, Dowsett M. A "quickscore" method for immunohistochemical semiquantitation: validation for oestrogen receptor in breast carcinomas. *J Clin Pathol* **1995**;48(9):876-8 doi 10.1136/jcp.48.9.876.

7. Bartley AN, Hamilton SR, Alsabeh R, Ambinder EP, Berman M, Collins E*, et al.* Template for Reporting Results of Biomarker Testing of Specimens From Patients With Carcinoma of the Colon and Rectum. *Archives of Pathology & Laboratory Medicine* **2013**;138(2):166-70 doi 10.5858/arpa.2013-0231-CP.

8. Welti J, Sharp A, Yuan W, Dolling D, Nava Rodrigues D, Figueiredo I*, et al.* Targeting Bromodomain and Extra-Terminal (BET) Family Proteins in Castration-Resistant Prostate Cancer (CRPC). *Clinical Cancer Research* **2018**;24(13):3149-62 doi 10.1158/1078-0432.Ccr-17-3571.

9. Welti J, Sharp A, Brooks N, Yuan W, McNair C, Chand SN*, et al.* Targeting the p300/CBP Axis in Lethal Prostate Cancer. *Cancer Discov* **2021**;11(5):1118-37 doi 10.1158/2159-8290.Cd-20-0751.

10. Workman P, Aboagye EO, Balkwill F, Balmain A, Bruder G, Chaplin DJ*, et al.* Guidelines for the welfare and use of animals in cancer research. *British Journal of Cancer* **2010**;102(11):1555-77 doi 10.1038/sj.bjc.6605642.

11. Drost J, Karthaus WR, Gao D, Driehuis E, Sawyers CL, Chen Y*, et al.* Organoid culture systems for prostate epithelial and cancer tissue. *Nat Protoc* **2016**;11(2):347-58 doi 10.1038/nprot.2016.006.
