# Supplementary material for: B7-H3 as a therapeutic target in advanced prostate cancer
Source: Eur Urol. Author manuscript; Available in PMC 2025 Aug 3. (PMC7617982; doi:10.1016/j.eururo.2022.09.004)
Supplement: Supplementary Tables [file EMS207305-supplement-Supplementary_Tables.docx]

**Supplementary Table S1: Patient characteristics**

| **98 patients with CRPC (72 patients had matching CSPC biopsies)** | |
| --- | --- |
| **Age at diagnosis (years)** |  |
| Median (95% confidence interval) | 62.4 (59.8-64.0) |
| **Stage at diagnosis (n)** |  |
| T1 | 1 |
| T2 | 9 |
| T3 | 42 |
| T4 | 23 |
| NR | 23 |
|  |  |
| N0 | 27 |
| N1 | 41 |
| NR | 30 |
|  |  |
| M0 | 35 |
| M1 | 59 |
| NR | 4 |
| **CSPC biopsy site** |  |
| Needle core | 58 |
| Radical prostatectomy | 5 |
| TURP  Bladder metastasis  Prostatic urethral metastasis | 7  1  1 |
| **CSPC histology** |  |
| Adenocarcinoma | 66 |
| Adenocarcinoma with neuroendocrine features | 1 |
| N/A | 5 |
| **CRPC histology** |  |
| Adenocarcinoma | 94 |
| Adenocarcinoma with neuroendocrine features | 4 |
| **Gleason score** |  |
| 10 | 10 |
| 9 | 46 |
| 8 | 9 |
| 7 | 15 |
| ≤6 | 7 |
| N/A | 11 |
| **Initial therapeutic intent** |  |
| Radical | 41 |
| Surgery | 11 |
| Radiotherapy | 37 |
| Palliative | 57 |
| **Site of metastatic biopsy** |  |
| Lymph node | 49 |
| Bone marrow trephine | 34 |
| Liver | 9 |
| Soft tissue | 5 |
| TURP | 1 |
| **Treatments prior to CRPC biopsy** |  |
| Androgen deprivation therapy | 98 |
| Abiraterone | 48 |
| Enzalutamide^b^ | 50 |
| Docetaxel | 82 |
| Cabazitaxel | 9 |

^a^20 patients received docetaxel during the hormone-sensitive setting

^b^1 patient received enzalutamide in the hormone-sensitive setting

TURP = transurethral resection of the prostate; CSPC = castration-sensitive prostate cancer; CRPC: castration-resistant prostate cancer

N/A = not applicable; NR = not recorded

**Supplementary Table S2: Antibody and IHC conditions**

| **Protein target** | **Retrieval buffer**  **(method)** | **Dilution**  **(time)** | **Detection** | **Controls** |
| --- | --- | --- | --- | --- |
| B7-H3  (#14058, clone D9M2L, Cell Signaling Technology) | pH8.1 Tris/EDTA  (microwave) | 1:100  (1 hour)  BioGenix i6000 autostainer (Launch Diagnostics) | DAKO EnVision Detection System (K4061; Dako, Agilent Technologies) | LNCaP non-silencing siRNA, LNCaP B7-H3 siRNA pellets and normal prostate. Rabbit IgG as negative control. |
| MMR  MSH2 (M3639, clone FE11)  MSH6 (M3646, clone EP49)  MLH1 (M3640, clone ES05)  PMS2 (M3647, clone EP51)  Dako, Agilent Technologies | pH8.1 Tris/EDTA  (Pressure cooker - Menapath Antigen Access Unit, Menarini diagnostics) | MSH2 – 1:50  MSH6 – 1:500  MLH1/ PMS2 – 1:100  (1 hour)  BioGenix i6000 autostainer (Launch Diagnostics) | DAKO EnVision Detection System (K4061; Dako, Agilent Technologies) | Appendix/ LNCaP (MSH2), Appendix/ skeletal muscle (MSH6), Appendix/ HCT116 (MLH1 and PMS2). Mouse and rabbit IgG as negative control. |
| ATM (ab32420, clone Y170, Abcam pl) | Target retrieval solution, pH9 (S236784-2, Dako, Agilent Technologies)  (Pressure cooker - Menapath Antigen Access Unit, Menarini diagnostics) | 1:400  (1 hour)  BioGenix i6000 autostainer (Launch Diagnostics) | DAKO EnVision Detection System (K4061; Dako, Agilent Technologies) | VCaP and GM01526 cell. Rabbit IgG as negative control. |
| PTEN (#9559, clone 138G6, Cell Signaling Technology) | pH6 citrate + tween  (microwave) | 1:250  (1 hour)  BioGenix i6000 autostainer (Launch Diagnostics) | ABC rabbit detection system (PK-6101, Vector Laboratories) | 22RV-1, PC3 and normal prostate. Rabbit IgG as negative control. |
| ARv7 (31-1109, clone RM7, RevMAb Biosciences) | pH8.1 Tris/EDTA  (microwave) | 1:500  (1 hour)  BioGenix i6000 autostainer (Launch Diagnostics) | DAKO EnVision Detection System (K4061; Dako, Agilent Technologies) | 22RV-1, VCaP, PC3. Rabbit IgG as negative control. |
| Synaptophysin  (NCL-L-SYNAP-299, clone 27G12, Leica Biosystems) | pH6 citrate  (microwave) | 1:200  (1 hour)  BioGenix i6000 autostainer (Launch Diagnostics) | DAKO EnVision Detection System (K4061; Dako, Agilent Technologies) | Pancreas and MCF7. |
| TP53 (#M7001, Dako, clone DO7, Agilent Technologies) | pH8.1 Tris/EDTA  (microwave) | 1:50  (1 hour)  BioGenix i6000 autostainer (Launch Diagnostics) | DAKO EnVision Detection System (K4061; Dako, Agilent Technologies) | DU145, PC3. Mouse IgG as negative control. |
| RB1  (#9309, clone 4H1, Cell Signaling Technology) | pH6 citrate  (Pressure cooker - Menapath Antigen Access Unit, Menarini diagnostics) | 1:1000  (1 hour)  BioGenix i6000 autostainer (Launch Diagnostics) | Novolink Polymer detection system  (RE7260, Leica Biosystems) | 22Rv1 non-silencing siRNA, 22Rv1 RB1 siRNA pellets and normal prostate. Mouse IgG as negative control. |
| Ki-67 (#M7240, clone MIB-1, Dako, Agilent Technologies) | pH6 citrate  (Pressure cooker - Menapath Antigen Access Unit, Menarini diagnostics) | 1:100  (1 hour)  BioGenix i6000 autostainer (Launch Diagnostics) | DAKO EnVision Detection System (K4061; Dako, Agilent Technologies) | Appendix. Mouse IgG as negative control. |
| p21 (#2947, clone 12D1, Cell Signaling) | Bond Epitope Retrieval Solution 1 (#AR9961, Leica Biosystems)  30 minutes | 1:100  (15 minutes)  (BOND RX, Leica Microsystems) | Bond Polymer Refine Kit (#DS9800, Leica Biosystems) | HeLa non-silencing siRNA, HeLa p21 siRNA pellets. Rabbit IgG as negative control. |
| p16 (#92803, clone D3W8G, Cell Signaling) | Epitope Retrieval Solution 2 (#AR9640, Leica Biosystems)  20 minutes | 1:300  (15 minutes)  (BOND RX, Leica Microsystems) | Bond Polymer Refine Kit (#DS9800, Leica Biosystems) | HeLa non-silencing siRNA, HeLa p16 siRNA pellets. Rabbit IgG as negative control. |
| CD56 (Clone 123C3, Agilent Technologies) | pH8.1 Tris/EDTA  (microwave) | 1:500 | DAKO EnVision Detection System (K4061; Dako, Agilent Technologies) | Mouse IgG as negative control. |
| Chromogranin A (Clone: ADK-A3, Agilent Technologies) | pH8.1 Tris/EDTA  (microwave) | 1:500 | DAKO EnVision Detection System (K4061; Dako, Agilent Technologies) | Mouse IgG as negative control. |

**Supplementary Table S3: DNA Damage Response (DDR) Targeted Panel Gene List**

| DNA Damage Response (DDR) Targeted Panel Gene List (n = 113) | | | | | |
| --- | --- | --- | --- | --- | --- |
| \| *AKT1* \| \| --- \| \| *AKT2* \| \| *ALK* \| \| *APC* \| \| *AR* \| \| *ARID1A* \| \| *ARID2* \| \| ***ATM*** \| \| ***ATR*** \| \| *ATRX* \| \| *AXIN1* \| \| *AXIN2* \| \| ***BARD1*** \| \| ***BLM*** \| \| *BRAF* \| \| ***BRCA1*** \| \| ***BRCA2*** \| \| *BUB1B* \| \| *CDH1* \| \| ***CDK12*** \| | \| *CDK4* \| \| --- \| \| *CDKN1B* \| \| *CDKN2A* \| \| ***CHEK1*** \| \| ***CHEK2*** \| \| *CTNNB1* \| \| *DDB2* \| \| *EGFR* \| \| *EPCAM* \| \| *ERBB2* \| \| *ERBB3* \| \| *ERBB4* \| \| ***ERCC2*** \| \| ***ERCC3*** \| \| ***ERCC4*** \| \| ***ERCC5*** \| \| ***ERCC6*** \| \| *EZH2* \| \| *FAM46C* \| \| ***FANCA*** \| | \| ***FANCB*** \| \| --- \| \| ***FANCC*** \| \| ***FANCD2*** \| \| ***FANCE*** \| \| ***FANCF*** \| \| ***FANCG*** \| \| ***FANCI*** \| \| ***FANCL*** \| \| ***FANCM*** \| \| *FGFR2* \| \| *FGFR3* \| \| *HNF1A* \| \| *HRAS* \| \| *JAK1* \| \| *JAK2* \| \| *KRAS* \| \| *MAP2K1* \| \| *MAP2K2* \| \| *MAP2K4* \| \| *MAP3K1* \| | \| *MAP4K3* \| \| --- \| \| *MDM2* \| \| *MET* \| \| ***MLH1**** \| \| ***MLH3**** \| \| *MRE11A* \| \| ***MSH2**** \| \| ***MSH3**** \| \| ***MSH6**** \| \| *MTOR* \| \| *MUTYH* \| \| *MYC* \| \| *MYD88* \| \| ***NBN*** \| \| *NF1* \| \| *NF2* \| \| *NFKBIA* \| \| *NOTCH1* \| \| *NOTCH2* \| \| *NRAS* \| | \| *NTRK1* \| \| --- \| \| ***PALB2*** \| \| *PDGFRA* \| \| *PIK3CA* \| \| *PIK3CG* \| \| *PIK3R1* \| \| *PMS2* \| \| *PRKDC* \| \| *PTEN* \| \| ***RAD50*** \| \| ***RAD51B*** \| \| ***RAD51C*** \| \| ***RAD51D*** \| \| *RB1* \| \| *RECQL4* \| \| *RET* \| \| *SMARCA4* \| \| *SMARCB1* \| \| *SPOP* \| \| *SRC* \| | \| *STK11* \| \| --- \| \| *TNFAIP3* \| \| *TNFRSF14* \| \| *TP53* \| \| *TSC1* \| \| *TSC2* \| \| *VHL* \| \| ***WRN*** \| \| *WT1* \| \| *XPA* \| \| *XPC* \| \| ***XRCC3*** \| \| *ZRSR2* \| |

DNA repair response genes in red

Mismatch repair genes denoted by *

**Supplementary Table S4: Cell lines**

| **Cell line** | **Supplier** | **Catalogue number** | **Media** | **Serum** |
| --- | --- | --- | --- | --- |
| 22Rv1 | ATCC | CRL-2505 | RMPI | FBS |
| VCaP | ATCC | CRL-2876 | DMEM | FBS |
| RWPE | ATCC | CRL-11609 | K-SFM | BPE/EGF |
| LNCaP | ATCC | CRL-1740 | RMPI | FBS |
| LNCaP95 | Dr Meeker/Dr Luo* | NA | RMPI^ | CSS |
| DU145 | ATCC | HTB-81 | DMEM | FBS |
| PC3 | ATCC | CRL-1345 | F12 HAM | FBS |
| PNT2 | Sigma-Aldrich | 95012613 | RPMI | FBS |

ATCC – American type culture collection, FBS – fetal bovine serum, CSS – charcoal stripped serum, * - LNCaP95 cells were kindly provided by Drs. Alan K Meeker and Jun Luo (Johns Hopkins University, Baltimore, Maryland, USA), NA – non-applicable, ^ - phenol red free, BPE/EGF - bovine pituitary extract and human recombinant epidermal growth factor.

**Supplementary Table S5: *nCounter PanCancer Immune Profiling Panel gene expression in SU2C and RMH Cohort (100 highest expressed genes)*** **CD276/B7-H3 highlighted*

| **SU2C Cohort** | | |  |  |  | **RMH Cohort** | | | | | |
| --- | --- | --- | --- | --- | --- | --- | --- | --- | --- | --- | --- |
|  | **Gene** |  | **Gene** |  | **Gene** |  | **Gene** |  | **Gene** |  | **Gene** |
| 1 | HLA-B | 35 | MAP2K2 | 69 | HLA-DPB1 | 1 | RPS6 | 35 | HLA-E | 69 | C1QB |
| 2 | RPS6 | 36 | VEGFA | 70 | LCP1 | 2 | UBC | 36 | VEGFA | 70 | STAT2 |
| 3 | CD63 | 37 | ISG15 | 71 | ANXA1 | 3 | CD63 | 37 | LAMP1 | 71 | BAX |
| 4 | UBC | 38 | CD46 | 72 | MAPK3 | 4 | CD81 | 38 | TAPBP | 72 | PIN1 |
| 5 | PPIA | 39 | LAMP1 | 73 | SPP1 | 5 | TUBB | 39 | IRAK1 | 73 | MIF |
| 6 | CD74 | 40 | CD276 | 74 | TNFRSF1A | 6 | CD9 | 40 | EWSR1 | 74 | HLA-DPB1 |
| 7 | HLA-C | 41 | CDH1 | 75 | PRKCD | 7 | PPIA | 41 | SPP1 | 75 | THBS1 |
| 8 | CD81 | 42 | ILF3 | 76 | TNFSF10 | 8 | HLA-B | 42 | STAT3 | 76 | HPRT1 |
| 9 | MIF | 43 | PIN1 | 77 | GUSB | 9 | CD74 | 43 | BCL2L1 | 77 | TNFRSF1A |
| 10 | CD99 | 44 | GTF3C1 | 78 | STAT3 | 10 | HLA-C | 44 | MAP2K2 | 78 | ST6GAL1 |
| 11 | CD9 | 45 | A2M | 79 | PSMD7 | 11 | TXNIP | 45 | CD47 | 79 | TFRC |
| 12 | CD24 | 46 | IFI27 | 80 | ENG | 12 | GPI | 46 | A2M | 80 | IFI27 |
| 13 | FN1 | 47 | LTBR | 81 | IRF3 | 13 | EPCAM | 47 | LCP1 | 81 | ANXA1 |
| 14 | HMGB1 | 48 | CD47 | 82 | NRP1 | 14 | HLA-DRA | 48 | LTBR | 82 | C1QA |
| 15 | APOE | 49 | LAMP2 | 83 | CKLF | 15 | HMGB1 | 49 | ANP32B | 83 | TOLLIP |
| 16 | CLU | 50 | TAPBP | 84 | ECSIT | 16 | COL3A1 | 50 | SMAD2 | 84 | ZC3H14 |
| 17 | TUBB | 51 | C1QB | 85 | PSMB10 | 17 | CD99 | 51 | HLA-DPA1 | 85 | AKT3 |
| 18 | HLA-A | 52 | IFITM1 | 86 | BCL2L1 | 18 | APOE | 52 | NRP1 | 86 | HRAS |
| 19 | IFITM2 | 53 | THY1 | 87 | CD44 | 19 | CD164 | 53 | CTSL | 87 | IFNGR1 |
| 20 | EPCAM | 54 | HLA-DPA1 | 88 | TMEFF2 | 20 | FN1 | 54 | IL6ST | 88 | IRF3 |
| 21 | HLA-DRA | 55 | IRAK1 | 89 | TNFRSF14 | 21 | HLA-A | 55 | CD276 | 89 | GUSB |
| 22 | CTSH | 56 | CTSL | 90 | STAT1 | 22 | CD59 | 56 | NFKBIA | 90 | ECSIT |
| 23 | COL3A1 | 57 | SDHA | 91 | HDAC3 | 23 | CD46 | 57 | SDHA | 91 | HDAC3 |
| 24 | GPI | 58 | BAX | 92 | FCER1G | 24 | PSMB7 | 58 | IFITM2 | 92 | IGF2R |
| 25 | TXNIP | 59 | HRAS | 93 | C3 | 25 | ITGB1 | 59 | GTF3C1 | 93 | SF3A3 |
| 26 | ANP32B | 60 | NFKBIA | 94 | ZC3H14 | 26 | TMEFF2 | 60 | IGF1R | 94 | IKBKB |
| 27 | PSMB7 | 61 | TYK2 | 95 | PSMB8 | 27 | CTSH | 61 | STAT1 | 95 | ITCH |
| 28 | APP | 62 | ALCAM | 96 | ABCF1 | 28 | ILF3 | 62 | POLR2A | 96 | RELA |
| 29 | CD59 | 63 | C1QA | 97 | TOLLIP | 29 | ALCAM | 63 | MAPK1 | 97 | YTHDF2 |
| 30 | EWSR1 | 64 | SERPING1 | 98 | STAT2 | 30 | CDH1 | 64 | ISG15 | 98 | ELK1 |
| 31 | ITGB1 | 65 | NOL7 | 99 | MRPS5 | 31 | LAMP2 | 65 | CTSS | 99 | LGALS3 |
| 32 | CD164 | 66 | LGALS3 | 100 | C1R | 32 | APP | 66 | PSMD7 | 100 | CREB1 |
| 33 | HLA-E | 67 | SIGIRR |  |  | 33 | CLU | 67 | PRKCD |  |  |
| 34 | C1QBP | 68 | RELA |  |  | 34 | C1QBP | 68 | CD44 |  |  |

**Supplementary Table S6: Summary table of genomic aberrations and mB7-H3 H-Score**

| **Patient number** | **Mutation status** | **ATM loss^a^** | **MMR loss^b^** | **PTEN loss^c^** | **mB7-H3** |
| --- | --- | --- | --- | --- | --- |
| 1 | SPOP p.F133L missense, AR p.F877L missense | No | No | Yes | 0 |
| 2 | KRAS p.G12D missense | Yes | No | No | 0 |
| 3 | TP53 p.R307 splice | No | No | No | 0 |
| 4 | PTEN p.T319* nonsense | Yes | No | Yes | 0 |
| 5 | BRAF p.K601E missense, PTEN p.D197 splice | No | No | Yes | 0 |
| 6 | TP53 p.N131K missense, TP53 p.M94_C96delinsI (inframe deletion insertion), MAP4K3 p.T105S missense | No | No | No | 0 |
| 7 | TP53 p.L194R missense | No | No | No | 0 |
| 8 | ERCC3 p.W374 nonsense, PTEN p.L140 nonsense, PIK3CA p.R4_E9del (inframe), SPOP p.F125C missense, MET p.Q318R missense | No | N/A | N/A | 5 |
| 9 | MSH2 HD, MSH6 HD | No | Yes | No | 5 |
| 10 | TP53 p.P151R missense | N/A | N/A | N/A | 5 |
| 11 | CTNNB1 p.533C missense SPOP p.F133V missense | N/A | N/A | N/A | 5 |
| 12 | PTEN HD, TP53 HD, PIK3CA p.E545A missense | No | No | Yes | 5 |
| 13 | PTEN HD | No | No | Yes | 5 |
| 14 | MSH6 p.214 nonsense | No | Yes | No | 10 |
| 15 | TP53 p.C141Y missense | No | No | Yes | 10 |
| 16 | CDK12 p.S582fs*56 frameshift (bi-allelic), PTEN p.E40* nonsense, SPOP p.F133L missense, CDK12 p.R882W missense. | No | No | No | 10 |
| 17 | No mutation detected | No | No | Yes | 15 |
| 18 | PIK3R1 p.Q475_splice | No | No | Yes | 15 |
| 19 | ATM p.W2960C missense | No | No | No | 20 |
| 20 | PTEN p.E235* nonsense, TP53 p.Y220C missense | No | No | Yes | 30 |
| 21 | PTEN HD | No | No | Yes | 30 |
| 22 | JAK1 p.C257Vfs*10 frameshift, JAK2 p.D620E missense | No | No | No | 35 |
| 23 | CDK12 p.Q1088Hfs*8 frameshift, MUTYH p.R203H missense | No | No | No | 35 |
| 24 | TP53 p.R273H missense | No | N/A | Yes | 40 |
| 25 | TP53 truncated p.Q317* | No | No | Yes | 40 |
| 26 | MSH2 HD | No | Yes | No | 40 |
| 27 | ATR p.L2417* nonsense, APC p.E1536* nonsense, SPOP p.F133L missense | No | No | No | 40 |
| 28 | FANCA HD, RB1 HD, ATM p.E2039K missense, | No | No | No | 50 |
| 29 | CDKN2A HD, PTEN HD, RB1 HD, SMARCB1 HD | No | No | No | 50 |
| 30 | MUTYH HD, RB1 HD, TP53 HD, BRAF p.K601E missense | No | No | N/A | 50 |
| 31 | TP53 p.R290H missense, TP53 p.R306* missense, NBN p.E658G missense | No | No | No | 60 |
| 32 | TP53 p.R213*; STK11 HD | N/A | N/A | N/A | 60 |
| 33 | ATM HD, APC p.A2122dup inframe insertion | Yes | No | No | 70 |
| 34 | WT1* p.C350* | N/A | N/A | N/A | 80 |
| 35 | BRCA2 HD | No | No | No | 85 |
| 36 | TP53 p.R342* missense | No | No | Yes | 90 |
| 37 | JAK p.1051* Nonsense and .S1056C missense | Yes | No | No | 90 |
| 38 | BRCA2 p.E1320* nonsense, CTNNB1 p.G34E missense, MSH6 p.G566* nonsense | N/A | No | N/A | 90 |
| 39 | BRCA2 p.L1768Rfs*5 frameshift | No | No | Yes | 95 |
| 40 | TP53 p.R337C missense | No | No | No | 100 |
| 41 | MLH3 p.R93* Nonsense | N/A | N/A | N/A | 100 |
| 42 | ARID1A fs* p.M2055Ifs*39 | Yes | No | No | 100 |
| 43 | No mutation detected | No | No | Yes | 100 |
| 44 | ARID1A fs* p.D1850Gfs*4; AR p.T878S missense | No | No | No | 100 |
| 45 | ATM p.Q2522* nonsense, ATM p.R2506_splice | Yes | N/A | No | 110 |
| 46 | CDK12fs p.K652Nfs*3 frameshift, CDK12 p.F986V missense | Yes | N/A | N/A | 115 |
| 47 | PTEN p.V258 splice, TP53 HD | No | No | No | 115 |
| 48 | MSH2 p.Y408Lfs*9 frameshift | No | Yes | Yes | 120 |
| 49 | BRCA2 p.T3085Nfs*26 frameshift, PTEN p.M270Nfs*28 missense | No | No | Yes | 125 |
| 50 | No mutation detected | No | No | Yes | 130 |
| 51 | BRCA2 HD | No | N/A | N/A | 130 |
| 52 | CDKN1B HD, TP53 HD | N/A | N/A | N/A | 130 |
| 53 | CDKN2A HD, PTEN HD | No | No | Yes | 130 |
| 54 | ATM HD | Yes | No | No | 140 |
| 55 | FANCLp.T372Nfs*13 frameshift | No | No | No | 140 |
| 56 | PTEN HD, TP53 HD | No | No | Yes | 150 |
| 57 | MSH6 p.I225fs*22 frameshift | No | Yes | No | 155 |
| 58 | ATM p.Y370* nonsense | Yes | N/A | No | 160 |
| 59 | BRCA2p.Q1089Sfs*10 frameshift ; RECQL4 p.377*nonsense | No | No | Yes | 160 |
| 60 | BRCA2 HD, APC p.R2431K missense | No | N/A | Yes | 165 |
| 61 | CDK4 p.K297fs*2 frameshift, TP53 p.S269N missense, ARID1A p.S1985F missense | No | No | Yes | 165 |
| 62 | No mutation detected | N/A | No | N/A | 170 |
| 63 | TP53 p.H258Qfs*44 frameshift | No | No | No | 175 |
| 64 | BRCA1 EX13 Dup frameshift | No | No | Yes | 180 |
| 65 | BRCA2 p.T2026fs*13 frameshift, FANCI p.F1043_splice, PTEN p.F520* nonsense, ERCC4 p.V756Afs*5 frameshift | No | No | Yes | 180 |
| 66 | PTEN p.R130* nonsense, TP53 p.N247D missense | No | No | Yes | 180 |
| 67 | JAK1 p.Q161_splice, TP53 p.Y234C missense | No | No | No | 185 |
| 68 | AR p.T878A missense | No | No | No | 190 |
| 69 | BRAF p.K601E missense | N/A | No | Yes | 190 |
| 70 | TP53 p.C176Y missense, STK11 HD | N/A | N/A | N/A | 195 |
| 71 | TP53 p.M133T missense | N/A | No | No | 200 |
| 72 | BRCA2 HD | No | N/A | Yes | 200 |
| 73 | BRCA2 HD, SPOP p.W131C missense, RB1 p.Q702 splice | No | No | No | 210 |
| 74 | PALB2 p.K75* nonsense, CDK12 p.A916G missense, ATM p.C2488Y missense | No | No | N/A | 220 |
| 75 | MSH2 p.K567Rfs*4 | No | Yes | N/A | 230 |
| 76 | No mutation detected | Yes | No | Yes | 230 |
| 77 | RAD51C HD, PTEN HD, TP53 p.R158H missense | No | No | Yes | 235 |
| 78 | TP53 p.E224D missense | No | No | No | 235 |
| 79 | BRCA2 p.C1200* nonsense, AR p.L702H missense, AR p.T878A missense, MLH3 p.S666_Q668del inframe deletion | No | No | No | 240 |
| 80 | ATM p.L1448* nonsense, ATM p.M56_splice | Yes | N/A | No | 240 |
| 81 | BLM p.R899* nonsense , CDKN1B p.E126* nonsense, NBN P700Qfs*13 frameshift | N/A | N/A | N/A | 245 |
| 82 | ATM p.V356_splice | Yes | N/A | No | 250 |
| 83 | CDK12 p.M816fs*5 frameshift, CDK12 p.F998_splice | No | N/A | N/A | 250 |
| 84 | ATM p.R2909I missense, MAP4K3 p.R83* nonsense | Yes | No | No | 250 |
| 85 | BRCA2 HD | N/A | No | N/A | 250 |
| 86 | ATM HD | Yes | No | Yes | 255 |
| 87 | ATM p.Y889*nonsense | Yes | N/A | N/A | 265 |
| 88 | PTEN HD, WRN HD, TP53 p.R273H missense | No | No | Yes | 265 |
| 89 | ATM p.P2842S missense, PTEN p.Y138Lfs*42 frameshift, TP53 p.P278L missense | No | No | Yes | 270 |
| 90 | No mutation detected | N/A | No | N/A | 270 |
| 91 | BRCA2 p.R2973Ifs*3 frameshift ,AR p.L702H missense | No | No | No | 270 |
| 92 | FANCA HD, FANCD2 HD, PTEN HD, AKT2 HD, TP53 p.G245S missense | No | N/A | N/A | 270 |
| 93 | ATM p.K1192Rfs*3 frameshift | Yes | N/A | No | 275 |
| 94 | BRCA2 HD, TP53 p.T125_splice | No | No | No | 280 |
| 95 | BRCA2 HD, AR p.L702H missense, PTEN p.L140* nonsense, PIK3CA p.R4_E9del inframe deletion | N/A | No | N/A | 280 |
| 96 | BRCA2 HD | No | N/A | No | 290 |
| 97 | NF1 p.Y2285* nonsense, TP53 p.S241F missense | No | No | Yes | 290 |
| 98 | TP53 p.Y220C missense | N/A | No | No | 300 |

^a^ ATM loss defined as nuclear ATM H-score 0

^c^ MMR status segregated by College of American Pathologists Criteria for biomarker reporting in colorectal carcinoma.

^b^ PTEN loss defined as cytoplasmic PTEN H-score $\leq$10

HD = homozygous deletion

*nonsense mutation

**Supplementary Table S7: Key genomic alterations of tumour biopsy and PDX model generated from the biopsy**

| Patient liver biopsy (adenocarcinoma) | | PDX CP327 |
| --- | --- | --- |
| Gene | Alteration present^a^ | Alteration Present^a^ |
| ARID2 | p.C382Ydf | YES |
| ARID2 | p.T426M | YES |
| FANCD2 | p.F925Sfs*17 | YES |
| FANCF | p.L162Dfs*103 | YES |
| MLH3 | p.L747V | YES |
| MTOR | p.D1278N | YES |
| TP53 | p.R196* | YES |
| MSH2 | No mutation (protein loss) | No mutation (protein loss) |
| MSH6 | No mutation (protein loss) | No mutation (protein loss) |

| Patient liver biopsy (adenocarcinoma) | | PDX CP341 |
| --- | --- | --- |
| Gene | Alteration present^a^ | Alteration present^a^ |
| ATM | p.R1466G (protein present) | YES (protein present) |
| TP53 | p.G245V | YES |
| ERCC3 | Deep deletion | YES |
| PTEN | Deep deletion (protein loss) | NO (protein loss) |
| RB1 | Deep deletion (protein present) | YES (protein present) |

| Patient lymph node biopsy (adenocarcinoma) | | PDX CP50 |
| --- | --- | --- |
| Gene | Alteration present^b^ | Alteration present^b^ |
| MRE11A | p.Y179 | YES |
| Chromosome 8 | Gain | YES |
| AR | Amplification | YES |
| ATM | No mutation (protein loss) | No mutation (protein loss) |

| Patient lymph node (adenocarcinoma with neuroendocrine differentiation) | | PDX CP142 |
| --- | --- | --- |
| Gene | Alteration present^b^ | Alteration present^b^ |
| TP53 | Y220C | YES |
| ERBB4 | S522L | YES |
| RB1 | No mutation (protein loss) | No mutation (protein loss) |

^a^Next generation sequencing

^b^Whole exome sequencing
