# Supplementary material for: B7-H3 as a therapeutic target in advanced prostate cancer
Source: Eur Urol. Author manuscript; Available in PMC 2025 Aug 3. (PMC7617982; doi:10.1016/j.eururo.2022.09.004)
Supplement: Supplementary Figures Legends [file EMS207305-supplement-Supplementary_Figures_Legends.docx]

**Supplementary Figure S1.** Overview of the clinical cohorts analysed by immunohistochemistry for B7-H3 protein expression, next-generation sequencing for tumour genomics, and clinical outcomes.

**Supplementary Figure S2.** Association between cytoplasmic and membranous B7-H3 protein expression with Spearman’s rank-correlation coefficient (r_s_).

**Supplementary Figure S3.**

Boxplots of B7-H3 (CD276) mRNA expression in PC in the **(A)** SU2C/PCF CRPC cohort and **(B)** ICR/RMH cohort comparing B7-H3 gene expression in tumours without DDR gene alteration (SU2C/PCF: n=167; ICR/RMH: n=68) and tumours with *BRCA1/2* mutation/homozygous deletion (SU2C/PCF: n=45; ICR/RMH: 20). Horizontal bars denote IQRs and medians. *P-*values calculated using Mann-Whitney U test. ICR/RMH = Institute of Cancer Research/Royal Marsden Hospital; IQR = Interquartile range; DDR = DNA damage response.

**Supplementary Figure S4.** Treatment history of patients from whom tumour biopsies were obtained for the generation of CP327, CP341, CP50 and CP142 PDXs and PDX-Os.

**Supplementary Figure S5.** Dose-response curves of DXd in PC cell lines **(A)** and PDX-Os **(B)** after 6 days of treatment. Data for 3 individual experiments and at least 3 technical replicates per experiment are shown.

**Supplementary Figure S6. (A, C, E)** Tumour volume over time in PDXs treated with DS-7300a (anti-B7-H3 ADC), parental anti-B7-H3 antibody, non-targeting IgG1-ADC, or vehicle obtained from a longitudinal mixed effect model with per mouse random intercept and slope. The median and standard error are presented. **(B,D,F)** Forest plots showing the results from the longitudinal mixed effect model for log-transformed tumour volume. Estimates and *P*-values refer to the interaction term of treatment arm and time indicating the difference in tumour volume slopes between each treatment arm and the vehicle arm.

**Supplementary Figure S7. (A)** Representative IHC for Ki67, B7-H3, p21, p16, and hematoxylin and eosin staining of end-of-treatment CP341 PDX tumours from mice treated with vehicle control (3.4 ml/kg) or DS-7300a (10 mg/kg). 100 µm scale bar. **(B)** Ki67 expression (% positive cells) in end-of-treatment CP341 PDX tumours. Tumour Ki67 in mice treated with DS-7300a was significantly lower than those treated with the non-targeting IgG1-ADC (10 mg/kg: *p<*0.001), the anti-B7-H3 antibody (3 mg/kg: *p<*0.001, 10 mg/kg: *p=*0.01), and the vehicle control (10 mg/kg: *p*=0.02). Bars represent maximum and minimums, interquartile ranges, and median. *P-*values were calculated using the unpaired Student’s t-test.
